# Biofortification of rice with lysine using endogenous histones

**DOI:** 10.1007/s11103-014-0272-z

**Published:** 2014-12-17

**Authors:** H. W. Wong, Q. Liu, S. S. M. Sun

**Affiliations:** 1 State Key Laboratory of Agrobiotechnology and School of Life Sciences, The Chinese University of Hong Kong, Shatin, N.T. Hong Kong, China; 2 Key Laboratory of Plant Functional Genomics of the Ministry of Education, College of Agriculture, Yangzhou University, Yangzhou, China; 3 Present Address: SCG90, Science Center, The Chinese University of Hong Kong, Shatin, N.T. Hong Kong, China

**Keywords:** Lysine, Biofortification, Rice (*Oryza sativa* L.), Histone, Food safety, Chalkiness

## Abstract

Rice is the most consumed cereal grain in the world, but deficient in the essential amino acid lysine. Therefore, people in developing countries with limited food diversity who rely on rice as their major food source may suffer from malnutrition. Biofortification of stable crops by genetic engineering provides a fast and sustainable method to solve this problem. In this study, two endogenous rice lysine-rich histone proteins, RLRH1 and RLRH2, were over-expressed in rice seeds to achieve lysine biofortification. Their protein sequences passed an allergic sequence-based homology test. Their accumulations in rice seeds were raised to a moderate level by the use of a modified rice glutelin 1 promoter with lowered expression strength to avoid the occurrence of physiological abnormalities like unfolded protein response. The expressed proteins were further targeted to protein storage vacuoles for stable storage using a glutelin 1 signal peptide. The lysine content in the transgenic rice seeds was enhanced by up to 35 %, while other essential amino acids remained balanced, meeting the nutritional standards of the World Health Organization. No obvious unfolded protein response was detected. Different degrees of chalkiness, however, were detected in the transgenic seeds, and were positively correlated with both the levels of accumulated protein and lysine enhancement. This study offered a solution to the lysine deficiency in rice, while at the same time addressing concerns about food safety and physiological abnormalities in biofortified crops.

**Electronic supplementary material:**

The online version of this article (doi:10.1007/s11103-014-0272-z) contains supplementary material, which is available to authorized users.

## Introduction

Rice is an important staple food, supplying 20 % of the world’s dietary energy, as well as serving as the primary food source of 17 Asian and Pacific, nine North and South American, and eight African countries (FAO 2004). It is also the sole stable food source in many developing countries (Pellett and Ghosh 2004) where food availability and diversity is limited (Sautter et al. 2006; Zhu et al. 2007). However, rice provides insufficient vitamin A, iron, and lysine, an essential amino acid, resulting in serious malnutrition in these countries (Sautter et al. 2006). Industrial supplementary and fortification programs have been proposed as remedial measures, but these methods are often not sustainable in developing countries because of chemical instability of supplements, costs, political instability, and the logistic challenge of reaching scattered populations (Sautter et al. 2006, Zhu et al. 2007; Mayer et al. 2008). Biofortification through agricultural biotechnology has been proposed as a more sustainable alternative, developing stable crops with enhanced nutritional value to fulfill the daily nutritional requirements of humans (Sautter et al. 2006; Zhu et al. 2007; Mayer et al. 2008; Hirschi 2009).

To biofortify rice with lysine, three major approaches can be used: (1) increase the accumulation of free lysine; (2) manipulate the seed storage proteins (SSPs); and (3) overexpress lysine-rich proteins in seeds. The two key enzymes in lysine biosynthesis, aspartate kinase (AK) and dihydrodipicolinate synthase (DHPS), are feedback-inhibited by lysine (Galili et al. 2002), so for the first approach efforts have been made to elevate lysine content by expressing lysine feedback-insensitive forms of these two enzymes in crops. For example, expression of native feedback-insensitive AK (*lysC*) from *E. coli* and DHPS (*dapA*) from *Corynebacterium glutamicum*, Falco et al. (1995) increased the lysine content up to five-fold in canola and soybean seeds. Huang et al. (2005) successfully doubled the lysine content in corn seeds by over-expressing lysine feedback-insensitive DHPS from *C. glutamicum* while reducing the accumulation of zein. Another strategy is to suppress the expression of lysine ketoglutarate reductase/saccharopine dehydrogenase (LKR/SDH), the key enzymes in the lysine degradation pathway, using antisense or RNA interference (RNAi) methods (Zhu and Galili 2004; Hournard et al. 2007). Synergistic manipulation of both lysine biosynthesis and catabolic enzymes could further enhance the free lysine levels in transgenic maize by up to 4,000 p.p.m. (Frizzi et al. 2008) and in rice by up to 60-fold (Long et al. 2012).

The discovery of the *opaque*-*2* (*o2*) mutant (Mertz and Bates 1964) in maize prompted the very different SSP approach to enhance lysine content in cereal crops. The *o2* mutation significantly reduced the levels of 22-kDa α-zein in corn, which was compensated by other lysine-rich proteins, thus increasing the lysine level (Mertz and Bates 1964; Schmidt et al. 1990; Segal et al. 2003). The retention of endogenous 22 and 19-kDa α-zeins in the rough ER of the maize mutants *floury2* (Coleman et al. 1997) and De*-*B30* (Kim et al. 2004) induced strong unfolded protein response (UPR) and enhanced the level of high-lysine ER chaperones and binding proteins, such as ER chaperone luminal binding protein (BiP). In rice, the knockdown of 13-kDa prolamin could elevate the total lysine content up to 56 % (Kawakatsu et al. 2010a) as a result of compensatory increases in lysine-richer glutelin, globulin, and BiP; however, it led to smaller protein bodies (PBs) with modified structures. Over-accumulation of BiP could also increase the total lysine content up to 2.9-fold (Kawakatsu et al. 2010b) but was accompanied by severe decreases in starch content and rice seed weight.

The identification of seed and endosperm-specific promoters in crop plants will facilitate the third approach. With these promoters, proteins with desired properties can be accumulated in crop seeds for biofortification. Examples in rice include the seed-specific overexpression of the artificial nutrient-rich protein *Asp*-*1* gene (Potrykus 2003) and the expression of a fusion gene encoding the winged bean lysine-rich protein and the rice lysine-rich glutelin 1 (GT1), which increased total lysine content in transgenic seeds by 58 % (Sun and Liu 2008). The lysine-rich protein approach has great potential for lysine biofortification, since the pool of protein-bound amino acids is larger than that of free amino acids in crop seeds (Galili and Amir 2013).

While research using these three approaches has led to fruitful progress, two concerns remain: (1) the potential allergenicity of candidate transgene products and (2) the occurrence of abnormalities in transgenic crops. Regarding allergenicity, the candidate genes used for biofortification are often foreign to the host crop (Shaul and Galili 1992; Falco et al. 1995; Huang et al. 2005) and may have unknown function (Yu et al. 2004), raising concerns for consumer acceptance and food safety (Weale 2010; Bawa and Anilakumar 2013). An historical example is the transfer of a methionine-rich protein gene from Brazil nut to soybean for methionine biofortification, resulting in an allergenic product (Altenbach et al. 1989; Nordlee et al. 1996). Unfortunately, the potential allergenicity of the transgenic product is often not considered in biofortification research.

The other concern is the frequent presence of physiological abnormalities in transgenic biofortified crops. Over-accumulation of free lysine in tobacco was shown to affect its vegetative growth and floral and seed development (Shaul and Galili 1992). Similar results were observed in free lysine-biofortified transgenic canola and soybean (Falco et al. 1995), which showed decreased germination rates. UPR (Urade 2007) was another abnormality in several important high-lysine maize mutants, including *o2*, *floury2*, and De*-*B30* (Coleman et al. 1997; Hunter et al. 2002; Kim et al. 2004). Over-expression or suppression of BiP also triggered strong UPR in transgenic rice seeds (Kawakatsu et al. 2010b; Wakasa et al. 2011). In these cases, UPR strongly affected host gene expression profiles, inducing the accumulation of ER chaperones (e.g., BiP) and other protein processing enzymes (e.g., protein disulfide isomerase, PDI) in the ER of seed cells and attenuating storage protein translation (Gething and Sambrook 1992; Urade 2007; Fanata et al. 2013), resulting in reduced protein content, abnormal PBs and protein storage vacuoles (PSVs), decreased grain weight and starch content, and increased chalkiness of the crop seeds. UPR and chalkiness were also observed in rice overexpressing the winged bean lysine-rich protein-rice GT1 fusion gene (Sun and Liu 2008; Yu 2008), in which two- to three-fold increases in BiP and PDI levels and abnormal PBs and PSVs were detected.

In this study, we aimed to generate transgenic rice biofortified with protein-bound lysine while also addressing food safety and plant physiology concerns. To address food safety and allergenicity, we first surveyed the GenBank database to identify rice endogenous proteins that were rich in lysine (>10 mol%) to lessen both the possible interference of a “foreign” protein (Chao and Krewski 2008) and ethical concerns. We then carried out sequence-based homology tests of these proteins against the databases of known allergens through AllergenOnline and Allermatch, as suggested by FAO and WHO (2001); this approach was recently shown to have about 94 % accuracy (Verma et al. 2011). Two endogenous histone proteins met our criteria. To avoid triggering physiological abnormalities by UPR and histone interference with normal seed physiology, we (1) carefully regulated the expression levels of the candidate proteins to enhance the lysine level in balance with those of other amino acids (Joint WHO/FAO/UNU Expert Consultation 2007) and (2) targeted the transgene protein products to seed PSVs for stable storage to avoid possible interference with other cellular functions. Through these strategies, we were able to generate transgenic rice lines with up to 35 % more lysine than the wild type (WT) and with no significant UPR detected.

## Materials and methods

### Identification of candidate proteins for lysine biofortification in rice seeds

We surveyed the GenBank database to identify potential protein candidates with the following traits: (1) endogenous to rice; (2) high lysine content (>10 mol%); (3) known function or with high homology to proteins of known function; (4) complete cDNA sequences; and (5) nonallergenicity, as determined by subjecting the protein sequences to 8-mer, 80-mer, and full FASTA searches in the allergen databases AllergenOnline and Allermatch for homology to known allergens, as suggested by the WHO/FAO guidelines for genetically modified foods (FAO and WHO 2001).

### Cloning of candidate genes and functional removal of potential nuclear localization signals

To clone *RLRH1* (GenBank:Os05g0113900) and *RLRH2* (GenBank:Os01g0502900), the primer pair A5NS (5′-GGGGATCCATGGACGTCGGCGTCGGCGG-3′) and A3 (5′-GGGAATTCCTAGGAGCGCGCCTGCTTC-3′) and the primer pair B5NS (5′-GGGGATCCATGGCGCCCAAGGCAGAG-3′) and B3 (5′-GGGAGCTCCTAGATCTCGCGGGAGGTGG-3′) were designed based on the respective cDNA sequences.

RT-PCR was carried out to clone the two candidate genes. Leaves of *Oryza sativa ssp. japonica* *cv.* 9983 were used to extract total RNA as described by Zheng et al. (1993). Each RT-PCR reaction used 1 μg total RNA sample and followed the protocol of SuperScript™ II reverse transcriptase (Invitrogen, Carlsbad, CA, USA). Platinum^®^ Taq DNA Polymerase High Fidelity (Invitrogen, Carlsbad, CA, USA) were used for second-strand synthesis. The PCR products were purified for T-vector ligation and subsequent DNA sequencing.

The potential nuclear localization signals (NLSs) of the two candidate proteins were predicted using PSORT (http://psort.hgc.jp/form.html). The cloned genes were amplified by PCR (Supplementary Fig. 5) using specific primers bearing mutations to change the amino acids in the predicted signals to either glycine or alanine and so alter the signal function (Supplementary Fig. 6).

### Vector construction and plant transformation

Vectors p1017, containing the regular rice GT1 promoter–GT1SP–GT1 terminator expression cassette in the super binary vector pSB130M (Sun and Liu 2008), and p1011, containing the modified rice GT1 promoter pmGT1, were provided by Prof. Q. Liu. pmGT1 was cloned from p1011 to replace the original GT1 promoter in p1017. In pA1, pA2, pB1, and pB2, BamHI and SacI were used to insert the target genes into vector p1017. NcoI and SacI were used in the remaining constructs.

All constructs were made with the super binary vector pSB130M. The second T-border set of the vector contains a hygromycin R selectable marker gene. The constructs were transformed into *Agrobacterium tumefaciens* EHA105 by the heat-shock method and then introduced via the *Agrobacterium* into primary calli derived from mature seeds of *O. sativa* ssp. *japonica cv.* 9983. Calli transformation, selection and regeneration were performed as previously described (Liu et al. 1998).

### Plant cultivation

Transformed rice plants were grown in a greenhouse for further analyses and identification, then propagated to the T2 generation in experimental fields at Yangzhou University, Jiangsu Province, China, with the approval of the Ministry of Agriculture, PRC. The field plots were randomly arranged, and T2 seeds were collected for further analyses.

### Confirmation of gene integration

Primers GT1S (5′-GAACAACACAATGCTGCGTC-3′) and A3S (5′-CTAGGAGCGCGCCTGCTTC-3′) were used for PCR screening of plants harboring *RLRH1* and primers GT1S and B3S (5′-CTAGATCTCGCGGGAGGTG-3′) were used for *RLRH2*. Seeds of positive lines were germinated to obtain materials for amino acid analysis (AAA), Southern blot, western blot, and transmission electron microscopy (TEM).

For Southern blot analysis, genomic DNA samples were extracted from green leaves using the CTAB method (Yu et al. 2005). For *RLRH1* lines, 20 μg genomic DNA from each sample was digested by NdeI (NEB) for 24 h, while in *RLRH2* lines, BamHI (NEB) was used. Electrophoresis, blotting, hybridization, and detection were carried out as described by Li et al. (2009) using gene-specific digoxigenin-labeled probes.

### Amino acid analysis

Seeds of WT and T2 lines harvested in the experimental fields were used for AAA. Rice samples without husks were ground to powder and dried overnight in a 55 °C oven to remove moisture. For each sample, two technical replicates of 0.01 g rice powder were weighed. Each sample was hydrolyzed with 1 mL 6 N HCl (H0636; Sigma, St. Louis, MO, USA) in Sarstedt 2-mL screw-cap tube, and 10 nmol L(+)-norleucine (140-07291; Wako Pure Chemicals, Osaka, Japan) was added. The samples were heated at 110 °C for 24 h, then the HCl was evaporated for 6 h at 65 °C. Dried samples were dissolved in 1 mL Na–S buffer (2 % sodium citrate, 1 % HCl, 0.1 % benzoic acid) and filtered with an Acrodisc^®^ 0.45 μm nylon membrane syringe filter (4426T; Pall Life Sciences, Port Washington, NY, USA) for injection and analyses using amino acid analyzer L8900 (Hitachi, Tokyo, Japan).

Data obtained from HPLC were normalized with the level of norleucine for each sample. Cysteine and methionine levels were excluded as they are unstable in hydrolysis and were difficult to detect. Fifteen amino acids were investigated, which included: aspartic acid, threonine, serine, glutamic acid, proline, glycine, alanine, valine, isoleucine, leucine, tyrosine, phenylalanine, histidine, lysine, and arginine.

### Antibodies production and western blot analysis

The amino acid sequences KPAKASKDKAAKSPKKQARS and QEAAHLARYNKKPIA were chosen to produce synthetic peptides and subsequently antibodies against the two histone proteins RLRH1 and RLRH2, respectively. Anti-BiP antibody was from Stressgen^®^ (SPA-818; Enzo Life Sciences, Inc., Farmingdale, NY, USA) while anti-PDI polyclonal antibodies were from our laboratory. For western blot analysis, 10–20 T2 seeds were randomly selected from each transgenic line, and their husks were removed. Total protein was extracted with buffer containing 0.125 M Tris, pH 7, 4 M urea, 4 % SDS, and 5 % β-mercaptoethanol. The proteins in the total protein extract were separated by size using the tricine SDS-PAGE system (Schagger and von Jagow 1987) and blotted onto PVDF membrane (BioRad, Hercules, CA, USA) using the Towbin buffer system (Towbin et al. 1979). The concentration of primary antibodies was 1:2,000 and of secondary antibodies 1:30,000 (A-3687; Sigma). Signals were detected using the Aurora™ kit (ICN Biomedicals, Costa Mesa, CA, USA).

### Transmission electron microscopy (TEM)

Immature (10-DAF) T2 seeds were harvested from independent transformants harboring WT, pA1, pA2, pA3, pA4, pB3, and pB4 constructs respectively for TEM analysis. Half of each immature seed was subjected to total protein extraction and western blot, while the other half was fixed at 4 °C overnight in a fixation solution (0.1 M Na_3_PO_4_, 0.1 % gluteraldehyde, 4 % paraformaldehyde, pH 7). After washing, it was dehydrated in an ethanol series (30, 50, and 70 % for 10 min each; then 85, 95, 100 and 100 % for 20 min each) and finally infiltrated with LR White (London Resin Company, UK) overnight at 4 °C. Polymerization of LR White was finished at 60 °C in gelatin capsules after 24 h. Ultrathin sections (70–80 nm) were cut using a Reichert UltracutS microtome (Leica Microsystems, Wetzlar, Germany) and mounted on formvar-coated copper grids.

Immunolabeling was performed by first incubating the samples in blocking solution (5 % BSA in 0.1 M Na_3_PO_4_ and 0.1 % Tween 20, pH 7) for 1.5 h and then in anti-sera (1:250 for RLRH1, 1:100 for RLRH2; both in blocking solution) for 1 h, followed by washing and incubating in secondary antibody solution containing goat anti-rabbit IgG immunogold reagent (EMS 25104 and 25109; Electron Microscopy Sciences, Hatfield, PA, USA; 1:750 for RLRH1, 1:600 for RLRH2; in blocking solution) for 45 min. After washing, the grids were counter-stained with uranyl acetate (2.5 %) and lead citrate solutions. Signals were observed in the cells of aleurone (with nuclei but very few starch granules) and endosperm (no nucleus but more starch granules) using an H7650 transmission election microscope with AMT XR40 side-mount CCD camera (Hitachi, Tokyo, Japan).

## Results

### Identification of endogenous rice genes encoding high-lysine proteins with low allergenic potential

We surveyed the GenBank database for rice proteins rich in lysine (>10 mol%) and carried out 8-mer, 80-mer, and full FASTA searches using the allergen databases AllergenOnline and Allermatch to the lysine-rich proteins’ sequence homology with known allergens according to the WHO/FAO guidelines for genetically modified food (FAO and WHO 2001). Two candidates, RLRH1 (NP_001054458) and RLRH2 (EAZ12048), were selected. Both showed high homology to the rice histone H2 family and were expressed at low levels in rice seeds (Supplementary Fig. 1). RLRH1 had a lysine content of 14.7 mol% and passed the 8-mer, 80-mer, and full FASTA searches (AllergenOnline results summarized in Supplementary Fig. 2). RLRH2 had higher lysine content (20.6 mol%) and passed the 8-mer and 80-mer searches, but had a marginal hit with a latex allergen (35.4 % identity) in the FASTA search (see Supplementary Fig. 3).

We cloned the two corresponding genes from total RNA of *japonica* rice *cv.* 9983 leaves by RT-PCR (Supplementary Fig. 4). The potential NLSs of the two candidate proteins were located using PSORT and modified by PCR (Supplementary Figs. 5 and 6, respectively) to (1) use as complements to compare their sub-cellular locations among the expressed constructs; (2) prevent the expressed histone proteins from re-entering the nuclei and potentially interfering with normal cell physiology and function; and (3) decrease the homology of RLRH2 to the latex allergen. The two modified proteins passed all three FASTA searches (AllergenOnline results summarized in Supplementary Figs. 7 and 8, respectively).

### Strategy of expressing the candidate genes

**Fig. 1:**
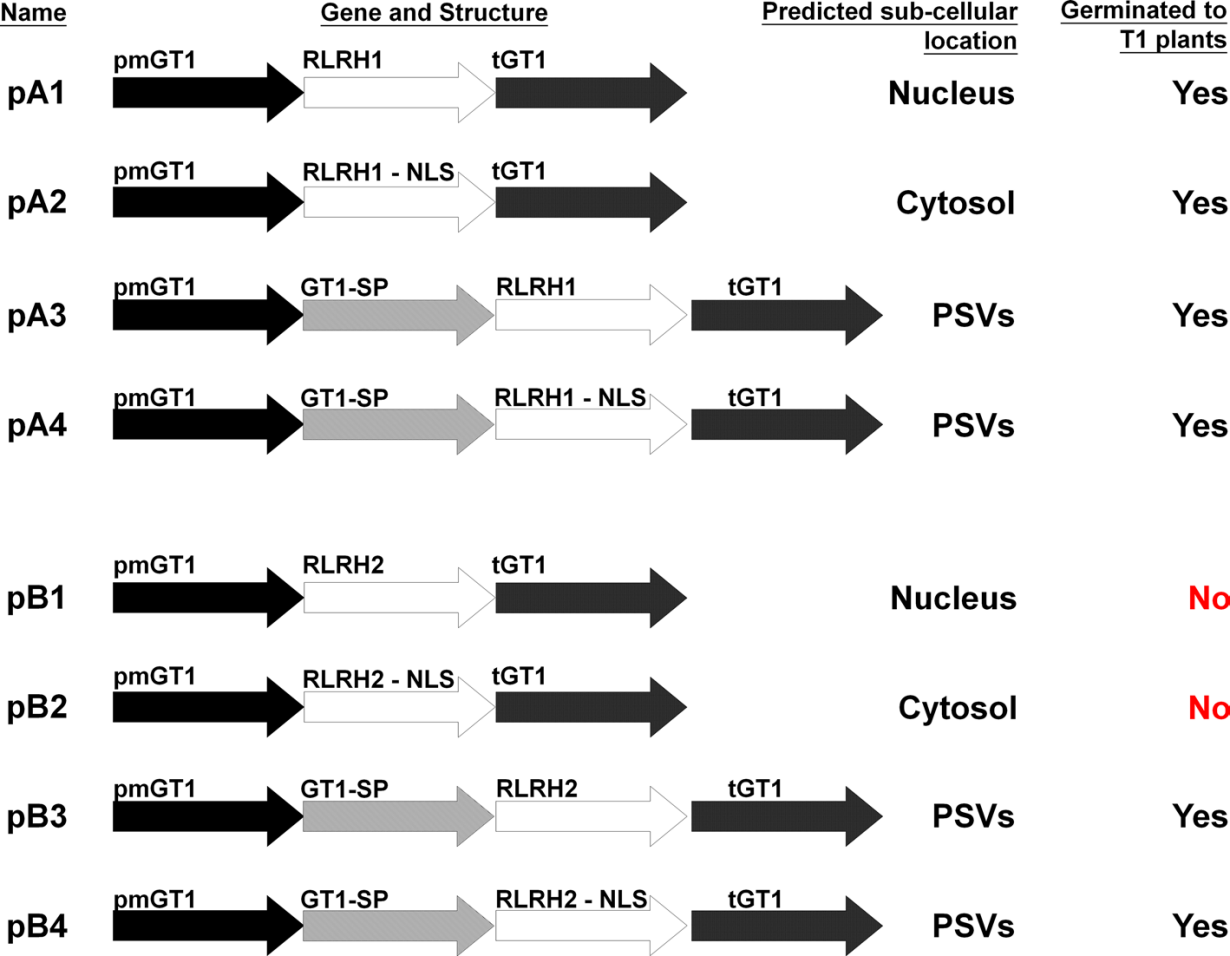
Constructs for the expression of *RLRH1* and *RLRH2* in transgenic rice seeds and their predicted sub-cellular locations. pmGT1, modified rice glutelin 1 promoter (1.3 kb); tGT1, glutelin 1 terminator; GT1-SP, glutelin 1 signal peptide; *RLRH1-NLS* and *RLRH2-NLS*, modified *RLRH1* and *RLRH2* in which predicted nuclear localization signal sequences were functionally removed

Figure 1 shows the structures of the gene constructs used in this study. Plasmids pA1 and pB1 contained a modified GT1 promoter (pmGT1), a GT1 3′ UTR (tGT1), and the cDNAs NP_001054458 (*RLRH1*) and EAZ12048 (*RLRH2*), respectively. The pmGT1 drives a moderate level of expression in the aleurone and endosperm cells of rice seeds compared to the original GT1 promoter (Liu 2002). It was used in this study to reduce the extent of UPR/ER stresses and chalkiness in transgenic rice seeds. The expressed histones of the two constructs carrying the targeting signals are expected to enter the nucleus. Plasmids pA2 and pB2 were similar to pA1 and pB1, except that the two histone genes were modified to remove the NLS function (Supplementary Figs. 5 and 6), and the resulting proteins were predicted to cytolocate to the cytoplasm. A GT1 signal peptide was included in constructs pA3, pA4, pB3, and pB4 to direct the expressed proteins to the ER. Similar to pA2 and pB2, the potential NLS function was also removed from *RLRH1* and *RLRH2* in constructs pA4 and pB4, respectively.

Rice plants regenerated from transformants with positive genomic PCR results were propagated to generate T1 seeds. At this stage, we found that the T1 seeds from constructs pB1 and pB2 failed to germinate, implying that the accumulation of RLRH2 or RLRH2-NLS in the nucleus and cytosol, respectively, might cause physiological abnormalities in the transgenic seeds. Positive transgenic plants containing the remaining six constructs were propagated to obtain T2 seeds.

### Amino acid analysis

The mature T2 seeds of 3–10 transgenic lines, each harboring constructs pA1-4 or pB3-4, were harvested in the fields for total AAA. For each construct, the lines with the maximum (high-lysine group; HLG) and minimum increases in lysine content (low-lysine group; LLG) were chosen for further study. The transgenic lines with highest increase of lysine in the HLG group (Table 1) came from constructs pA3 (35 % increase) and pB4 (24 % increase), suggesting that targeting protein into PSVs may positively affect lysine enhancement in rice seeds. At the same time, we detected decreases in glutamate, leucine, phenylalanine, and tyrosine, and, to a lesser extent, aspartate, isoleucine, and valine. In contrast, increased contents of glycine, histidine, proline, serine, and threonine were observed in most of the transgenic lines.

**Table 1:** Amino acid contents of high-lysine group (HLG) transgenic lines of rice

	WT (mg/g protein)	% difference from WT					
		pA1-High	pA2-High	pA3-High	pA4-High	pB3-High	pB4-High
Asx	*90.00* *±* *3.63*	0.00 ± 1.57	−2.91 ± 1.87	−0.75 ± 1.23	0.02 ± 4.53	−6.47 ± 2.91	−0.64 ± 0.64
Thr	*30.13* *±* *0.66*	3.81 ± 1.42	6.05 ± 0.54	7.77 ± 0.13	2.81 ± 2.60	6.05 ± 2.23	6.12 ± 0.82
Ser	*39.10* *±* *1.91*	5.19 ± 2.71	5.46 ± 3.84	5.33 ± 0.44	0.35 ± 6.83	6.68 ± 3.64	0.23 ± 0.25
Glx	*156.12* *±* *7.47*	−1.72 ± 0.19	−2.25 ± 1.19	−8.49 ± 0.32	−4.13 ± 0.29	−4.62 ± 0.24	−9.18 ± 0.43
Pro	*37.96* *±* *1.38*	−1.29 ± 2.32	−0.49 ± 3.83	5.04 ± 2.30	32.21 ± 2.40	48.52 ± 0.76	29.84 ± 3.08
Gly	*42.39* *±* *1.01*	4.51 ± 0.40	4.32 ± 0.13	11.70 ± 0.29	4.84 ± 0.05	4.03 ± 0.28	2.27 ± 0.22
Ala	*47.84* *±* *2.28*	0.02 ± 0.42	−0.02 ± 0.18	2.93 ± 0.46	0.33 ± 0.13	−1.16 ± 0.30	−0.93 ± 0.15
Val	*48.71* *±* *1.85*	−0.38 ± 0.64	−2.76 ± 0.17	−1.89 ± 0.05	−2.05 ± 0.43	−3.85 ± 0.27	−3.70 ± 0.50
Ile	*33.22* *±* *1.55*	−2.37 ± 0.66	−3.56 ± 0.58	−3.28 ± 0.39	−3.50 ± 0.36	−4.75 ± 0.27	1.26 ± 0.11
Leu	*73.21* *±* *4.57*	−4.31 ± 0.26	−4.82 ± 0.29	−8.99 ± 0.19	−7.00 ± 0.05	−7.53 ± 0.12	−6.70 ± 0.45
Tyr	*22.04* *±* *2.41*	−4.51 ± 0.78	−2.80 ± 1.47	−13.95 ± 0.72	−8.87 ± 0.60	−8.20 ± 0.74	−1.98 ± 0.38
Phe	*50.09* *±* *2.82*	−1.43 ± 0.08	−3.21 ± 0.19	−8.50 ± 0.85	−4.89 ± 0.15	−3.79 ± 0.15	−3.96 ± 0.51
His	*24.04* *±* *0.51*	3.23 ± 0.12	4.97 ± 0.48	11.99 ± 0.92	1.97 ± 0.39	1.66 ± 2.02	−1.63 ± 3.59
Lys	*35.46* *±* *1.17*	9.46 ± 0.11	10.98 ± 0.37	**35.65** ± 0.31	10.65 ± 0.07	7.49 ± 1.66	**24.39** ± 1.33
Arg	*70.99* *±* *2.10*	−0.31 ± 0.61	5.26 ± 0.95	2.29 ± 0.44	−2.19 ± 1.32	−1.90 ± 0.50	2.25 ± 0.12
Total	*801.29*						

Data were corrected for norleucine as an internal standard in each sample and are mean ± SD from duplicated samples. The amino acid content of wild-type (WT) seeds (in italics) and the percent change in transgenic lines are shown. Data in bold represent the two highest lysine elevations from the pA3 and pB4 constructs, respectively

*Asx* asparagine and aspartic acid, *Glx* glutamine and glutamic acid

Similarly, in the LLG (Table 2), we observed changes in the contents of some amino acids but to much lesser degrees. Interestingly, one pA1 line experienced a decrease in lysine (5 %). However, amino acids such as tyrosine, leucine, glutamate, alanine, and phenylalanine, which were decreased in most other transgenic lines, all increased in this line. These interesting results, together with the data from HLG and LLG, suggested that systematic changes in the contents of these amino acids may be closely linked to the expression of the candidate proteins and the level of lysine elevation.

**Table 2:** Amino acid contents of low-lysine group (LLG) transgenic lines

	WT (mg/g protein)	% Changes from WT					
		pA1-Low	pA2-Low	pA3-Low	pA4-Low	pB3-Low	pB4-Low
Asx	*90.00* *±* *3.63*	−0.76 ± 0.51	−2.45 ± 2.83	−0.97 ± 0.36	−2.58 ± 3.85	−3.99 ± 0.92	−0.64 ± 0.63
Thr	*30.13* *±* *0.66*	−4.29 ± 1.43	2.43 ± 1.65	1.40 ± 0.01	−0.71 ± 0.15	0.03 ± 0.60	−3.40 ± 0.33
Ser	*39.10* *±* *1.91*	−2.94 ± 0.74	3.26 ± 3.08	−2.16 ± 0.12	−1.59 ± 4.98	−0.11 ± 1.42	−4.13 ± 0.86
Glx	*156.12* *±* *7.47*	1.91 ± 0.48	2.13 ± 0.11	−1.75 ± 0.32	0.88 ± 0.38	−3.22 ± 0.61	−2.14 ± 0.48
Pro	*37.96* *±* *1.38*	−0.41 ± 2.06	6.76 ± 4.95	29.26 ± 1.12	28.52 ± 4.89	38.21 ± 2.77	46.70 ± 10.19
Gly	*42.39* *±* *1.01*	−1.36 ± 0.48	1.89 ± 0.44	2.24 ± 0.08	1.26 ± 0.05	−0.03 ± 0.14	−0.26 ± 0.67
Ala	*47.84* *±* *2.28*	1.08 ± 0.09	−0.54 ± 0.03	0.36 ± 0.30	0.32 ± 0.15	−0.74 ± 0.07	−1.06 ± 0.42
Val	*48.71* *±* *1.85*	0.57 ± 1.27	0.27 ± 0.77	−2.00 ± 1.30	−0.48 ± 0.44	−2.28 ± 0.43	−1.41 ± 0.37
Ile	*33.22* *±* *1.55*	−0.19 ± 0.28	−1.58 ± 0.73	−3.73 ± 0.75	−3.11 ± 0.48	−2.05 ± 0.24	−2.60 ± 1.19
Leu	*73.21* *±* *4.57*	1.68 ± 0.81	−2.41 ± 0.90	−3.77 ± 0.02	−3.38 ± 0.41	−4.03 ± 0.06	−4.81 ± 1.10
Tyr	*22.04* *±* *2.41*	6.82 ± 0.32	−6.15 ± 0.76	−4.00 ± 0.30	−3.59 ± 0.71	−1.24 ± 0.24	−6.64 ± 1.72
Phe	*50.09* *±* *2.82*	−0.13 ± 0.55	−0.87 ± 1.71	−3.62 ± 0.46	−3.50 ± 0.69	−0.42 ± 0.10	−2.62 ± 1.30
His	*24.04* *±* *0.51*	−2.37 ± 3.28	−1.63 ± 3.34	−1.60 ± 0.90	−2.62 ± 0.19	−1.33 ± 3.98	−2.47 ± 0.23
Lys	*35.46* *±* *1.17*	−5.24 ± 3.22	0.44 ± 1.95	2.31 ± 0.65	−0.97 ± 0.75	−1.03 ± 0.78	0.50 ± 0.56
Arg	*70.99* *±* *2.10*	−0.12 ± 1.83	−2.88 ± 0.38	−1.35 ± 1.19	−3.48 ± 1.04	0.61 ± 0.78	−3.22 ± 0.10
Total	*801.29*						

Data were corrected for norleucine as an internal standard in each sample and are mean ± SD from duplicated samples. The amino acid content of wild-type (WT) seeds (in italics) and the percent change in transgenic lines are shown presented

*Asx* asparagine and aspartic acid, *Glx* glutamine and glutamic acid

We also investigated whether the altered amino acid profiles of our transgenic rice lines could still fulfill the WHO amino acid requirements (Joint WHO/FAO/UNU Expert Consultation 2007). Among the amino acids tested, lysine was deficient in the WT seeds (Table 3), while the contents of lysine and other essential acids in pA3-HLG and pB4-HLG, the two transgenic lines with highest lysine levels, fulfilled the WHO requirements.

**Table 3:** Comparison of essential amino acid contents of wild-type (WT), transgenic rice, and the World Health Organization (WHO) 2007 standards

Essential amino acid contents				
	1	2	3	4
	WT (mg/g protein)	WHO requirements (mg/g protein)	pA3-HLG (mg/g protein)	pB4-HLG (mg/g protein)
Histidine	24.04	15	26.92	23.65
Isoleucine	33.24	30	32.13	33.64
Leucine	73.21	59	66.63	68.30
Phy + Tyr	72.13	38	64.80	69.71
Threonine	30.13	23	32.47	31.97
Valine	48.71	39	47.79	46.90
Lysine	**35.46**	45	48.10	44.11

The nutrient data in columns 1, 3 and 4 were based on total amino acid analysis of WT, pA3-HLG, and pB4-HLG strains, respectively. Data in column 2 are WHO 2007 standards. The datum that does not meet the WHO requirements is in bold

### Phenotyping of transgenic plants in HLG and LLG

Southern blots revealed that the target genes were incorporated into the genomes of HLG and LLG transgenic lines. In most of the HLG lines, the copy numbers of the target gene were higher than those in the corresponding LLG lines (Supplementary Fig. 9). The candidate genes were also detected in the WT because they are endogenous in rice.

**Fig. 2:**
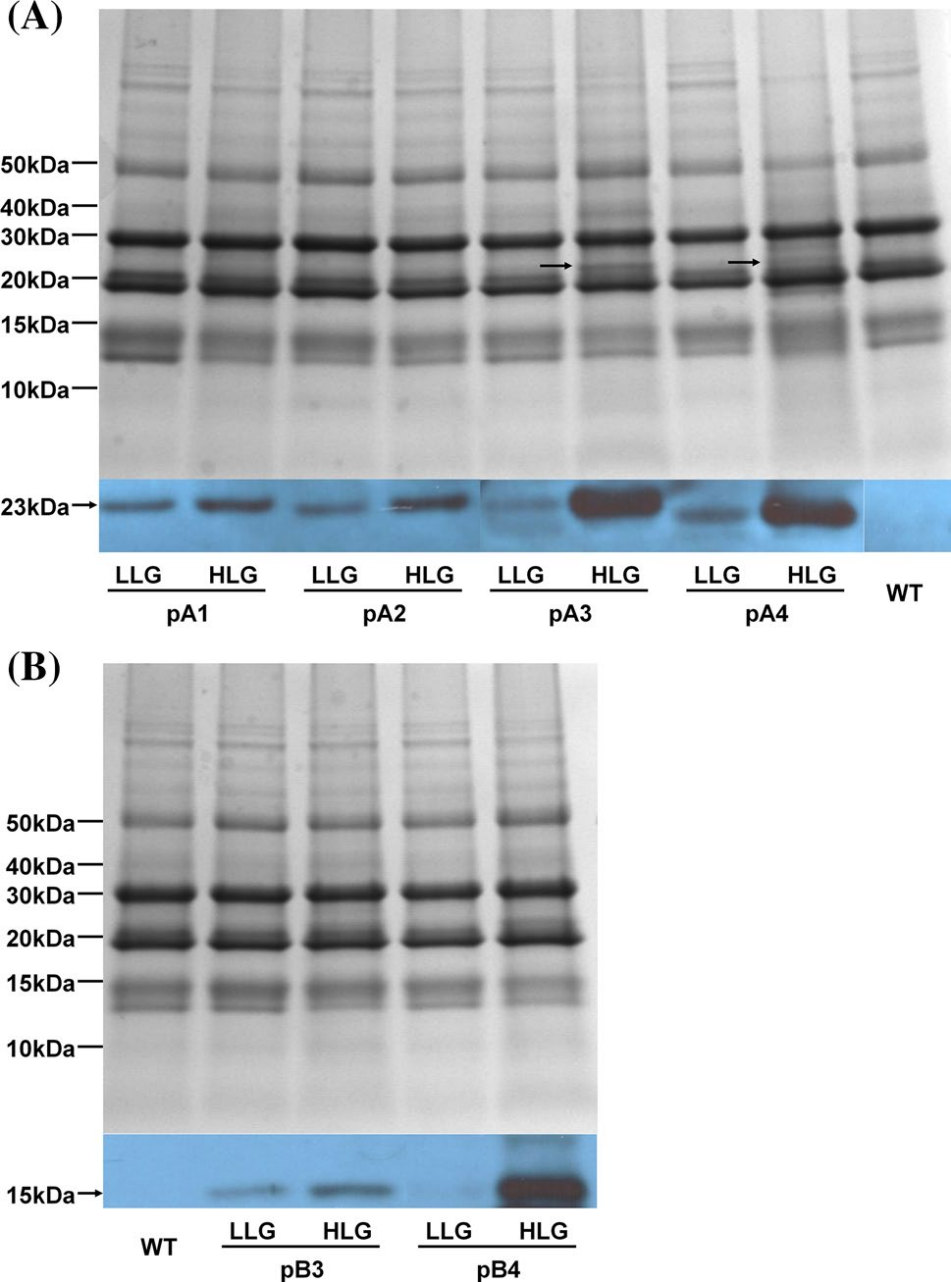
Western blots showing lysine enhancement caused by the accumulation of RLRH1, RLRH1-NLS, RLRH2, and RLRH2-NLS in transgenic seeds. **a** Accumulation levels of RLRH1 or RLRH1-NLS compared to wild type (WT). **b** Accumulation levels of RLRH2 or RLRH2-NLS compared to WT. Coomassie blue-stained protein gels (*in grey*) was included for reference in both **a** and **b**

All transgenic lines accumulated more of the two target proteins in the HLG than in the corresponding LLG (Fig. 2a, b, western blot in blue). Even within the HLG, a greater target protein accumulation corresponded to higher lysine content (Fig. 2a, b). Extra bands that corresponded in size to RLRH1 or RLRH1-NLS could also be observed in the total protein profiles of transgenic lines showing strong western-blot signals (arrows in Fig. 2a protein gel). We did not observe extra bands corresponding to RLRH2 or RLRH2-NLS, because this size coincides with endogenous storage proteins.

### Subcellular localization of expressed candidate proteins

Western blot results (Supplementary Fig. 1) showed that both RLRH1 and RLRH2 were expressed as nuclear proteins at a very low level in rice seeds. TEM revealed that both RLRH1 (Supplementary Fig. 10a) and RLRH2 (Supplementary Fig. 10f) were present in the nucleoplasm of the aleurone cells of WT seeds but not in other parts of the aleurone nucleus, and no signal was found in other sub-cellular compartments, including the cytosol, PBs, and PSVs, of the aleurone and endosperm cells. We were therefore not surprised that, without any targeting components, the over-expressed RLRH1 protein in pA1 rice was also located only in nucleoplasm of aleurone cells, as in the WT (Supplementary Fig. 10b and Supplementary Table 1).

**Fig. 3:**
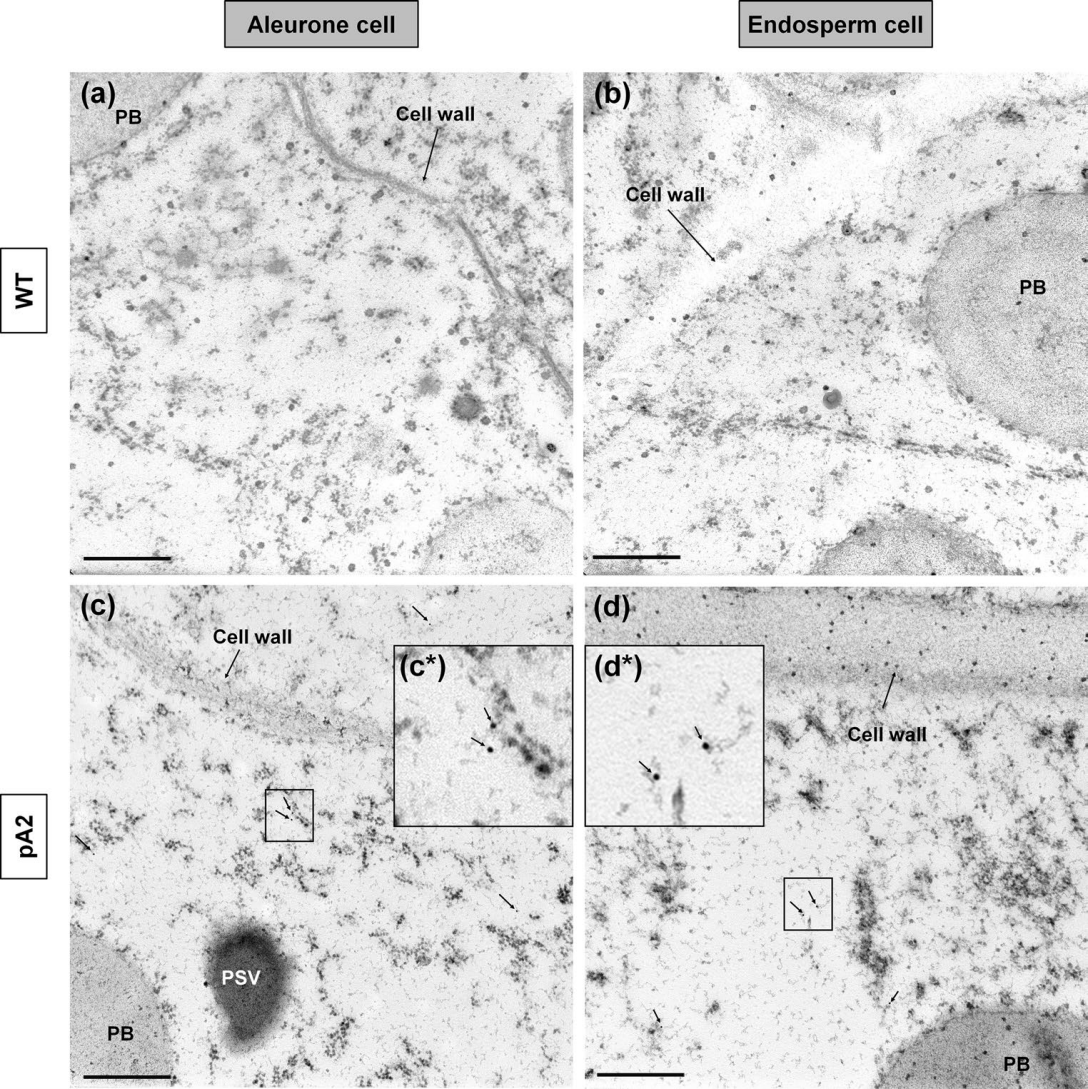
Transmission electron microscopy of the cytosols of rice seed cells. RLRH1-NLS accumulated in the cytosols of aleurone (**c**) and endosperm (**d**) cells of transgenic rice seeds harboring the pA2 construct, but not in the cytosol of aleurone (**a**) and endosperm (**b**) cells of wild type. Immunogold particles with 10 nm diameter were used. *Black arrows* indicate immunolabeled RLRH1-NLS. *Bar,* 500 nm. The *insets* with *asterisks* are enlargements of the *boxed areas*

In pA2 rice, the potential NLS of the *RLRH1* gene was mutated. TEM results from pA2 transgenic seeds indicated that the protein signals were located both in the nucleoplasm (Supplementary Fig. 10c) and cytosol of the aleurone cells (Fig. 3c) and in the cytosol of the endosperm cells (Fig. 3d). While the nucleoplasm signal came from endogenous RLRH1, as in the WT, the cytosolic signal in transgenic samples harboring pA2 probably originated from the over-expressed RLRH1-NLS protein, as supported by the absence of this signal in the cytosol of WT aleurone (Fig. 3a) and endosperm (Fig. 3b) cells. These results suggested that we successfully identified and eliminated the NLS function in *RLRH1* and changed the subcellular localization of the modified proteins.

**Fig. 4:**
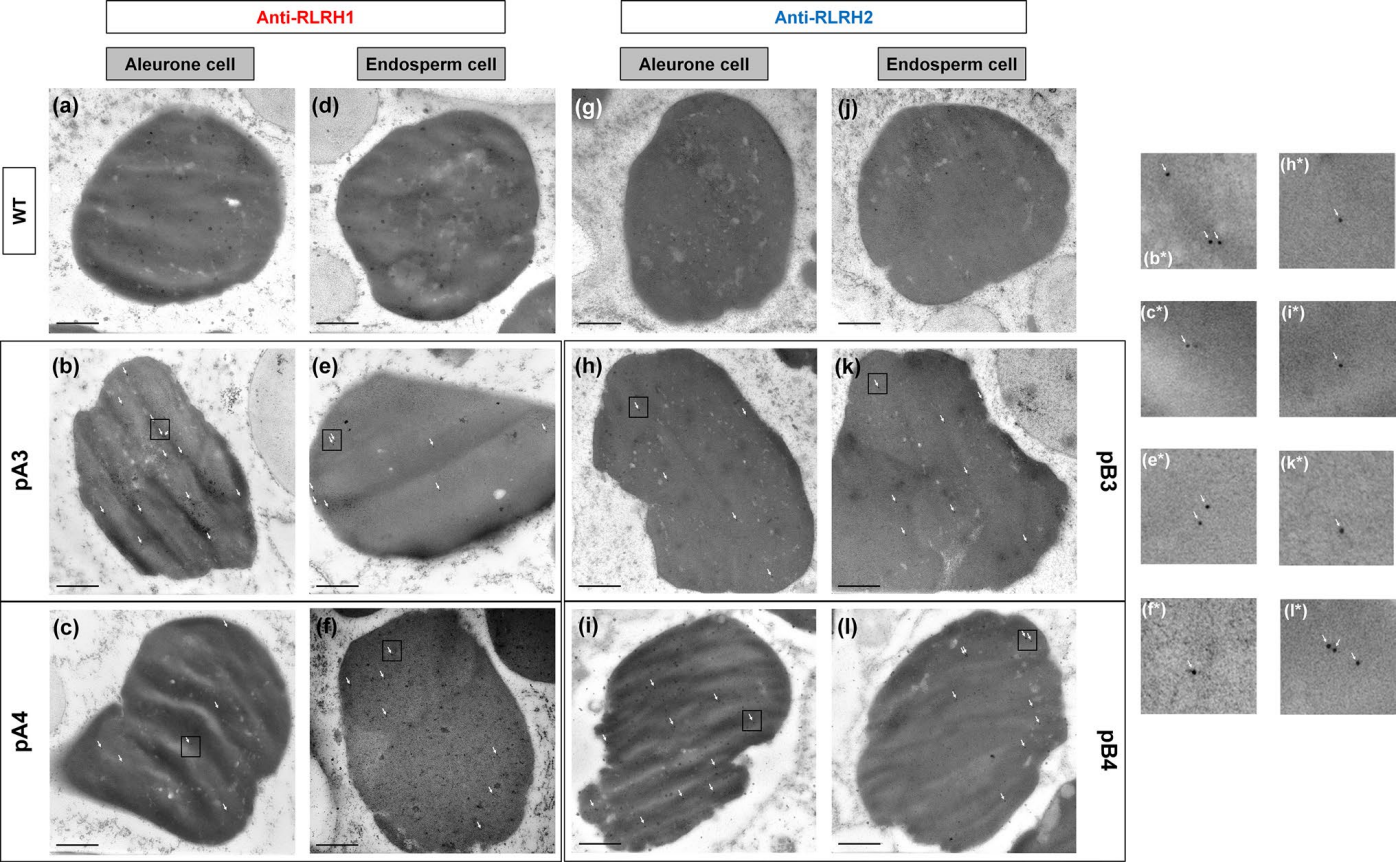
Expressions of *RLRH1* and *RLRH2* in protein storage vacuoles (PSVs) in the aleurone and endosperm cells of wild-type (WT) transgenic rice seeds. **a**, **d**, **g**, and **j** PSVs in WT cells labeled by gene-specific antibodies; **b**, **e** PSVs in cells of pA3. **c**, **f** PSVs in cells of pA4. **h**, **k** PSVs in cells of pB3. **i**, **l** PSVs in cells of pB4. Immunogold particles with 6 nm diameter were used. *White arrows* indicated immunolabeled candidate proteins. *Bar,* 500 nm. The *insets* with *asterisks* are enlargements of the *boxed areas*

In constructs pA3, pA4, pB3, and pB4, a GT1 signal peptide was added to the target gene sequence to direct the expressed protein to the ER. The TEM images showed that the majority of the immunolabeling signals were found in the PSVs of aleurone and endosperm cells in all these constructs (Fig. 4), indicating that the GT1 signal peptide targeted the expressed proteins into PSVs. Some signals were also observed in the nucleoplasm of the aleurone cells (Supplementary Fig. 10d, e, g, and h) because of endogenous background expression. Supplementary Table 1 summarizes the subcellular locations of the detected immunolabels in the aleurone and endosperm cells of different transgenic lines in comparison with WT.

### Observation for physiological abnormalities

**Fig. 5:**
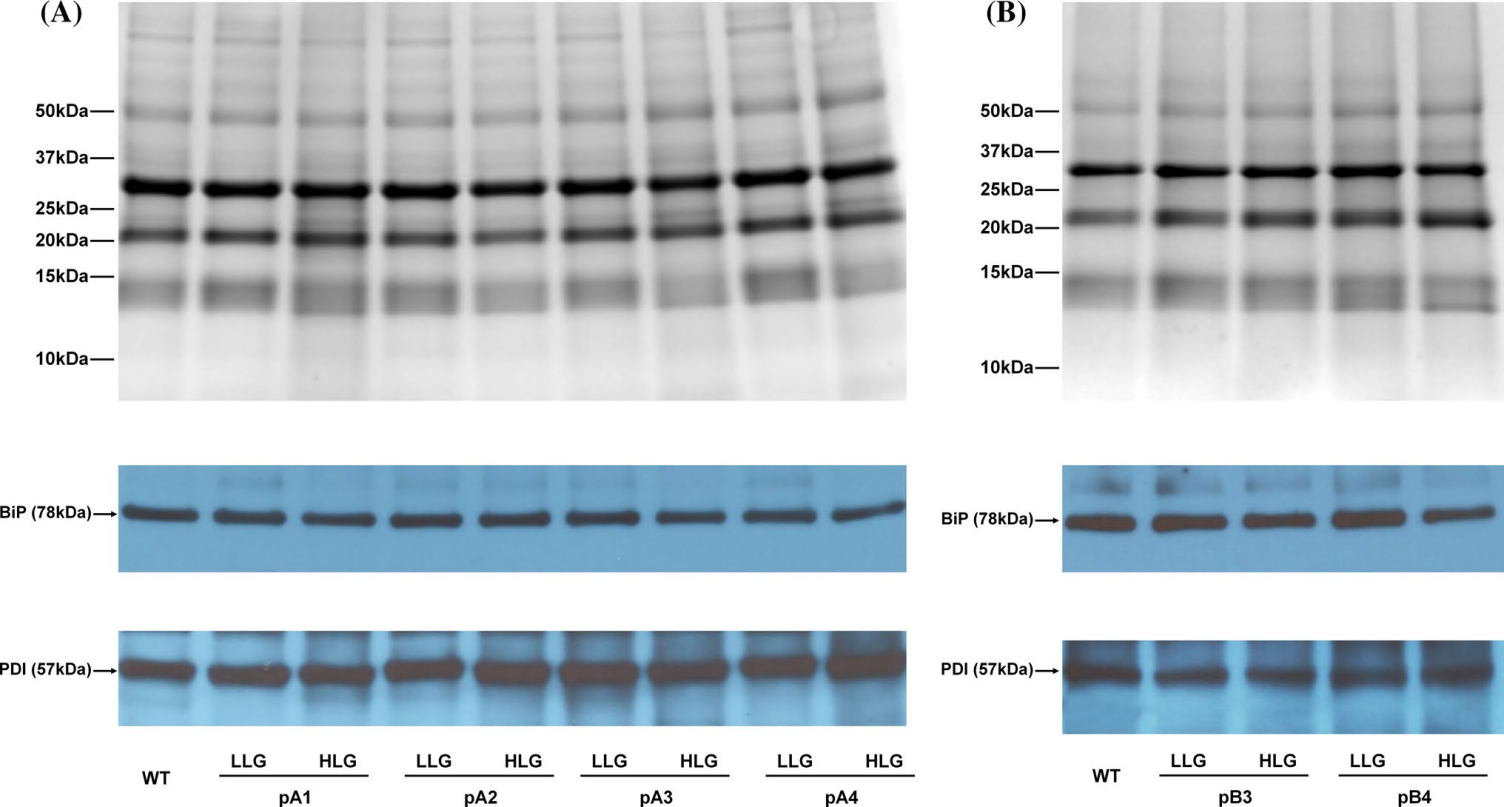
Accumulation levels of BiP and PDI in T2 seeds of *RLRH1* (**a**) and *RLRH2* (**b**) transgenic rice lines in comparison with the wild type (WT). The same amounts of total protein from the WT and *RLRH1* (**a**) and *RLRH2* (**b**) transformants were used for comparison. The concentration of anti-BiP antibody was 1:2,000 while the concentration of anti-PDI antibody was 1:5,000. Coomassie blue-stained protein gels (*in grey*) was included for reference in both **a** and **b**

Two key signatures of the occurrence of UPR are the increases in the level of ER chaperones and protein processing enzymes such as BiP and PDI (Hunter et al. 2002; Kim et al. 2004; Sun and Liu 2008; Yu 2008), and the appearance of abnormal PBs and PSVs (Coleman et al. 1997; Sun and Liu 2008; Yu 2008; Wakasa et al. 2011). In this study, however, we did not observe any abnormalities in these organelles of transgenic aleurone and endosperm cells by TEM (Supplementary Figs. 11 and 12). Western blot analysis using anti-BiP and anti-PDI antibodies showed that the amount of BiP and PDI in transgenic lines in HLG and LLG was not notably different from that in the WT (Fig. 5). Thus, by using the pmGT1 promoter to drive expression of the candidate genes, UPR was largely avoided.

To investigate whether the accumulation of RLRH1 or RLRH1-NLS and RLRH2 or RLRH2-NLS in transgenic rice seeds affected their germination, the rates of germination and seedling morphology of T2 seeds from HLG and LLG were compared to those of the WT seeds. The germination rates of the T2 seeds harboring different constructs were all over 90 %, similar to or somewhat higher than that of WT seeds (Supplementary Table 2). Seedling morphology was also similar to that of the WT, with no apparent abnormalities or retarded growth (Supplementary Fig. 13).

**Fig. 6:**
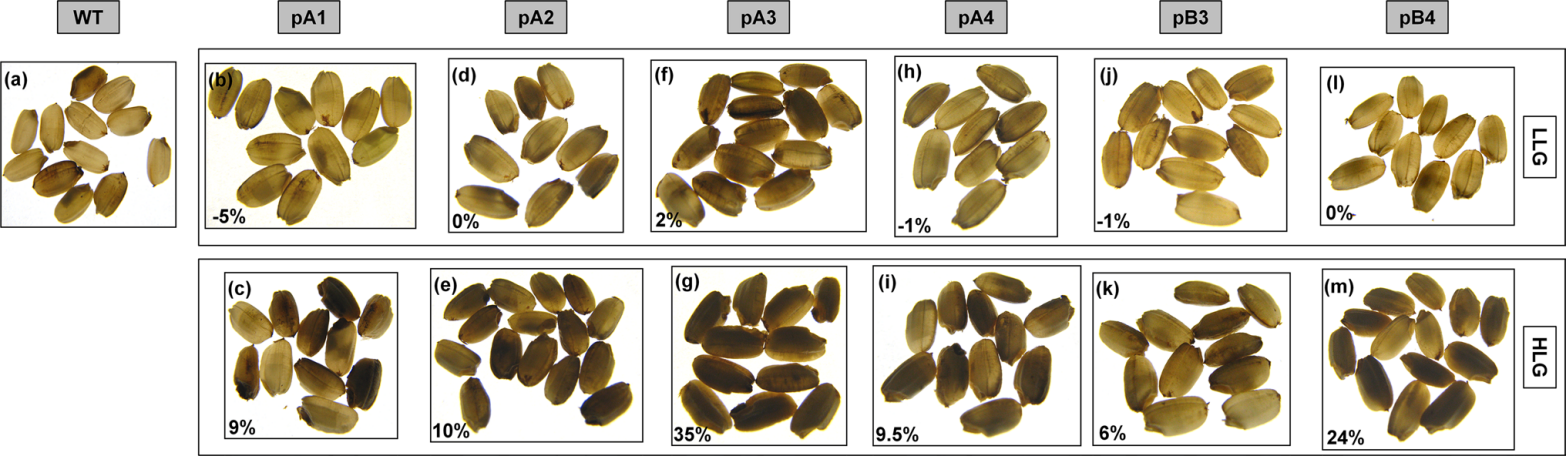
Degree of chalkiness in seeds of wild type (WT) and transgenic high-lysine group (HLG) and low-lysine group (LLG) lines. Mature seeds of WT and T2 transgenic lines bearing different constructs were observed under white transmitted light to observe the degree of chalkiness. One LLG line (*bottom row*) and one HLG line (*top row*) from each construct were selected for observation. The percentage change of lysine is shown at the *bottom left corner*

Among the T2 rice seeds, however, different degrees of chalkiness were observed. When examined with a light box, like the WT seeds (Fig. 6a), the transgenic seeds of LLG (same lines as in Table 2) were mostly translucent (Fig. 6b, d, f, h, j, l). Seeds in the HLG (same lines as in Table 1), on the other hand, showed different degrees of turbidity (chalkiness) that positively corresponded to their levels of protein accumulation and lysine enhancement (Fig. 6c, e, g, i, k, m). Interestingly, chalkiness was also found in the lysine-enhanced lines of pA1 and pA2, in which the RLRH1 and RLRH1-NLS proteins accumulated in the nucleus and cytosol, respectively, but did not enter the ER. Our results suggest that UPR is not directly linked to the degree of chalkiness, while the accumulation of lysine-rich protein in transgenic seeds did.

## Discussion

### RLRH1 and RLRH2 are good candidates for lysine biofortification

Although genetic engineering has been suggested as a tool in crop improvement, relatively few products have been released to market, in part because of concerns about GM food safety, including allergenicity, and the ethics of expressing exogenous genes in organisms. Also, many previous studies have reported physiological abnormalities in the nutrient-improved crops. We believed that through careful selection and expression of candidate transgenes, these concerns and risks could be addressed.

In this study, *RLRH1* and *RLRH2* were chosen as candidate transgenes because their gene products are native housekeeping histone proteins in rice seeds with very high lysine content and very low allergenic potential based on a known allergenic sequence-based homology test. The two candidate genes are relatively small, so cloning and manipulation was convenient. By careful regulation of their expressions and targeting of their protein products, we were able to avoid the occurrence of UPR and plant growth interference. To our knowledge, this report represents the first successful example of accumulating endogenous lysine-rich histone proteins specifically in crop seeds for lysine bio-fortification. Although an *Arabidopsis* histone gene, *AtHTA1*, driven by universal promoters had been expressed in *Arabidopsis* (Tenea et al. 2009) and rice (Zheng et al. 2009), those transgene studies were designed to enhance *Agrobacterium*-mediated transformation efficiency.

### Lysine biofortification triggers systemic changes in amino acid composition

The AAA results revealed that the level of lysine in our biofortified rice seeds was markedly increased to meet the recommended level of WHO/FAO. Among different transgenic lines harboring different constructs, the levels of lysine enhancement correlated with the target protein concentrations in the transgenic seeds.

We also observed some correlation between lysine elevation and changes in the contents of several other amino acids; these changes tended to be synchronous when the amino acids belonged to the same biosynthesis pathways. For example, we detected decreases in the contents of both leucine and valine, which are commonly synthesized using pyruvate as starting material. Similar patterns were observed in the decreases of both tyrosine and phenylalanine. The contents of both proline and histidine, which use glutamate as a starting material, and those of glycine and serine (3-phosphoglycerate) also increased simultaneously.

Systemic change in amino acid content is a common phenomenon in crop seeds with lysine elevation, e.g., in maize (Hunter et al. 2002; Segal et al. 2003; Huang et al. 2005) and rice (Kim et al. 2013). Although the change patterns were not identical in every case because of different strategies for lysine elevation, these studies shared common changes in some of the amino acids. Both threonine and glycine increased with lysine levels, while leucine and glutamate/glutamine decreased. These trends were also observed in our study, implying that the contents of these four amino acids may correlate closely to that of lysine in crop seeds.

Differences in amino acid content between RLRH proteins and rice major storage proteins (glutelin, accounting for 60–80 %, and prolamin, 20–30 % of the total proteins in rice seeds) may also contribute to some of the changes in amino acid content in transgenic seeds. For example, RLRH proteins have a lower content of glutamine/glutamate (5.77 mol% in RLRH1 and 10.78 mol% in RLRH2) when comparing to glutelin (15.63 mol% in GT1, 16.16 % in glutelin 2, and 15.2 mol% in glutelin 3) and prolamin (19.87 mol% in 13 kDa prolamin and 14.18 mol% in 10 kDa prolamin). Tyrosine content in RLRH proteins is also lower when compared to those in major storage proteins (2.56 and 1.96 mol%, respectively, in comparison to glutelin (3.5–3.8 mol%) and prolamin (4.5–5.2 mol%)). Nevertheless, this factor alone cannot explain the changes of all amino acids, e.g. proline.

### *RLRH1* and *RLRH2* were expressed and deposited in the predicted sub-cellular compartments

Protein targeting was chosen in this study to lessen the potential interference of the introduced proteins with normal physiological functions. The constructs pA1, pA2, pB1, and pB2 were designed to test the effect and efficacy of this strategy. We observed significant inhibition in the germination of T1 seeds harboring pB1 and pB2, suggesting that nucleus and cytoplasm of the rice seeds are not good locations for the over-accumulation of histone proteins. A recent study on the cytotoxicity of over-expressed histone in budding yeast revealed that excess histone may change the chromatin structure, bind to RNA inside the cells, saturate various types of histone modification enzymes, and disturb normal gene expression profiles (Singh et al. 2010), providing clues to why the seeds of pB1 and pB2 lines could not germinate.

TEM imagery of rice seeds harboring the pA2 construct showed that the storage location of RLRH1 was changed from the nucleus to the cytoplasm, demonstrating that substituting glycine and alanine into the predicted NLS can effectively stop its function, preventing the candidate protein from entering the nucleoplasm.

In transgenic seeds harboring constructs pA3, pA4, pB3, and pB4, immunolabeling signals were located extensively in PSVs, with only some background expression in nuclei, revealing that the GT1 signal peptide effectively directed the target proteins to PSVs for stable storage. The normal seed germination rate and seedling stage of T1 plants harboring pB3 and pB4 further revealed that histone accumulation in the PSVs of seed cells did not adversely affect these processes.

Through TEM observation, we found no abnormalities of the PBs and PSVs, nor in other cellular compartments in the aleurone and endosperm cells. Furthermore, the levels of BiP and PDI in transgenic seeds was not notably higher than in our previous studies (Sun and Liu 2008; Yu 2008). In addition, the seed germination rate and morphology of T2 seedlings were similar to those of the WT. These results implied that expression of the candidate histones in rice did not trigger observable UPR. Our results provide the first example of the biofortification of rice seeds with balanced essential amino acids, including lysine, through genetic engineering with a native high-lysine protein that passed allergenic sequence checks while also preventing the occurrence of UPR and abnormalities in the sub-cellular structures of aleurone and endosperm.

### Possible causes of chalkiness

Endospermic chalkiness is a change of seed endosperm from translucent to starchy white and is commonly found in genetically-modified cereal crops. In maize, chalkiness of three well-known mutants, *o2*, *floury 2* and De*-*B30*, were suggested to be closely related to UPR (Hunter et al. 2002; Kim et al. 2004; Urade 2007). Mutations in the signal peptides of their SSPs (α-zeins) caused the retention and accumulation of immature proteins in the ER. The “stresses” thus incurred resulted in attenuation of protein biosynthesis, reducing protein accumulation in ER and alternation of the morphology of protein storage organelles, so as to maintain the ER homeostasis (Urade 2007; Fanata et al. 2013). In consequence, the protein content in these seeds was reduced, and the organization of starch granules and protein storage organelles in endosperm changes, which cause the chalkiness phenotype. In rice, over-expression or suppression of BiP could also trigger chalkiness (Wakasa et al. 2011). ER stress, changed BiP levels, and chalkiness are thought to be closely related. Previous studies by our group (Sun and Liu 2008; Yu 2008) showed that over-expression of lysine-rich proteins in rice seeds, driven by the original 1.8-kb GT1 promoter and signal peptide, might have induced UPR with observable changes including: (1) elevated BiP and PDI levels (two- to three-fold); (2) abnormal PBs and PSVs; and (3) chalkiness in rice seeds.

In this study, our results revealed that chalkiness occurred in transgenic lines with strong protein accumulation and lysine enhancement. However, strong BiP and PDI elevation and PB and PSV abnormalities were not observed in these seeds. Furthermore, chalkiness occurred in the transgenic seeds regardless of whether the candidate proteins were expressed in the nucleus or cytosol (for example, the pA1 and pA2 constructs, respectively). Therefore, the chalkiness effect was unlikely to be caused by UPR. More importantly, the degree of chalkiness may correlate with the levels of protein and lysine accumulation, which has not been previously reported.

A recent transcriptome study (Liu et al. 2010) revealed that chalkiness in cereal seeds is, in fact, a quantitative genetic trait that involves complicated gene networks for carbohydrate metabolism, transcription, signal transduction, cell defense, redox homeostasis, and protein syntheses/degradation. When these networks are altered, chalkiness is easily triggered. Lysyl-tRNA synthetase, an enzyme important for lysine incorporation into proteins during translation, is involved in these networks. We are tempted to suggest, based on our current study, that the increase in protein-bound lysine may have stressed this enzyme with a high demand for its activity during seed filling, causing the enzyme to become limiting and triggering chalkiness.

Chalkiness is undesirable for cereal seed appearance and has been associated with the high lysine trait in many lysine-biofortified transgenic crops. Despite our efforts, we were unable to remove or reduce it in this study, further demonstrating the complexity of its occurrence. However, our lysine-biofortified transgenic rice with enhanced nutritional value can be processed, for example as rice flour, to make various products such as pastries and rice noodles, because the lysine availability is little affected by cooking or processing (Rutherfurd et al. 2012). It can also be used for animal feed.

### Future perspectives

With the HLG rice lines, we are planning field trials and propagation to achieve homozygous lines for future food-safety assessment and other experiments, such as in vitro simulated gastric and duodenal digestion to test the digestibility of the candidate histone proteins. The amount of candidate proteins in the aleurone and endosperm fractions can also be measured using these seeds. Mouse feeding tests are planned to study the nutritional and physiological effects of the rice on animals.

## Electronic supplementary material

Below is the link to the electronic supplementary material.

Supplementary material 1 (DOCX 11259 kb)
