# Supplementary material for: Biofortification of rice with lysine using endogenous histones
Source: Plant Mol Biol. 2014 Dec 17;87(3):235–48. doi: 10.1007/s11103-014-0272-z (PMC4302240; doi:10.1007/s11103-014-0272-z)
Supplement: Supplementary file 1 — Supplementary material 1 (DOCX 11259 kb) [file 11103_2014_272_MOESM1_ESM.docx]

**Supplementary Materials**

**
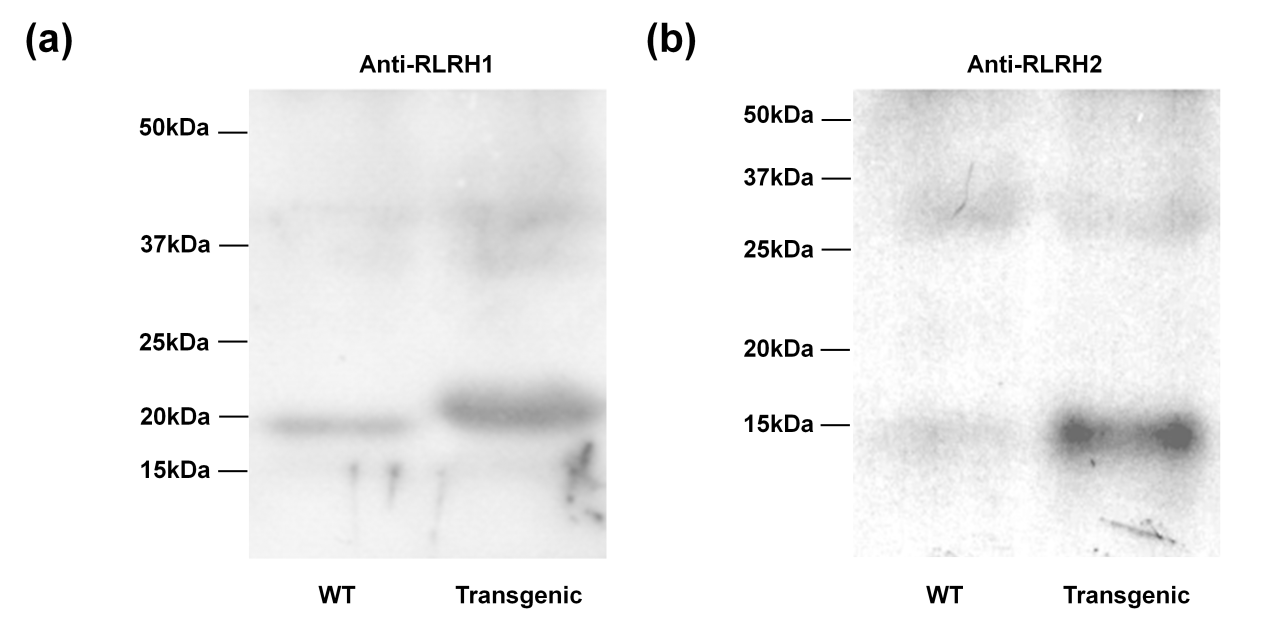
**

**Supplementary Fig. 1** Expression of *RLRH1* and *RLRH2* in rice seeds. The expression levels of *RLRH1* (**a**) and *RLRH2* (**b**) in WT and transgenic seeds were detected by their specific antibodies at a concentration of 1:2,000. The apparent sizes were 20 and 15 kDa, respectively


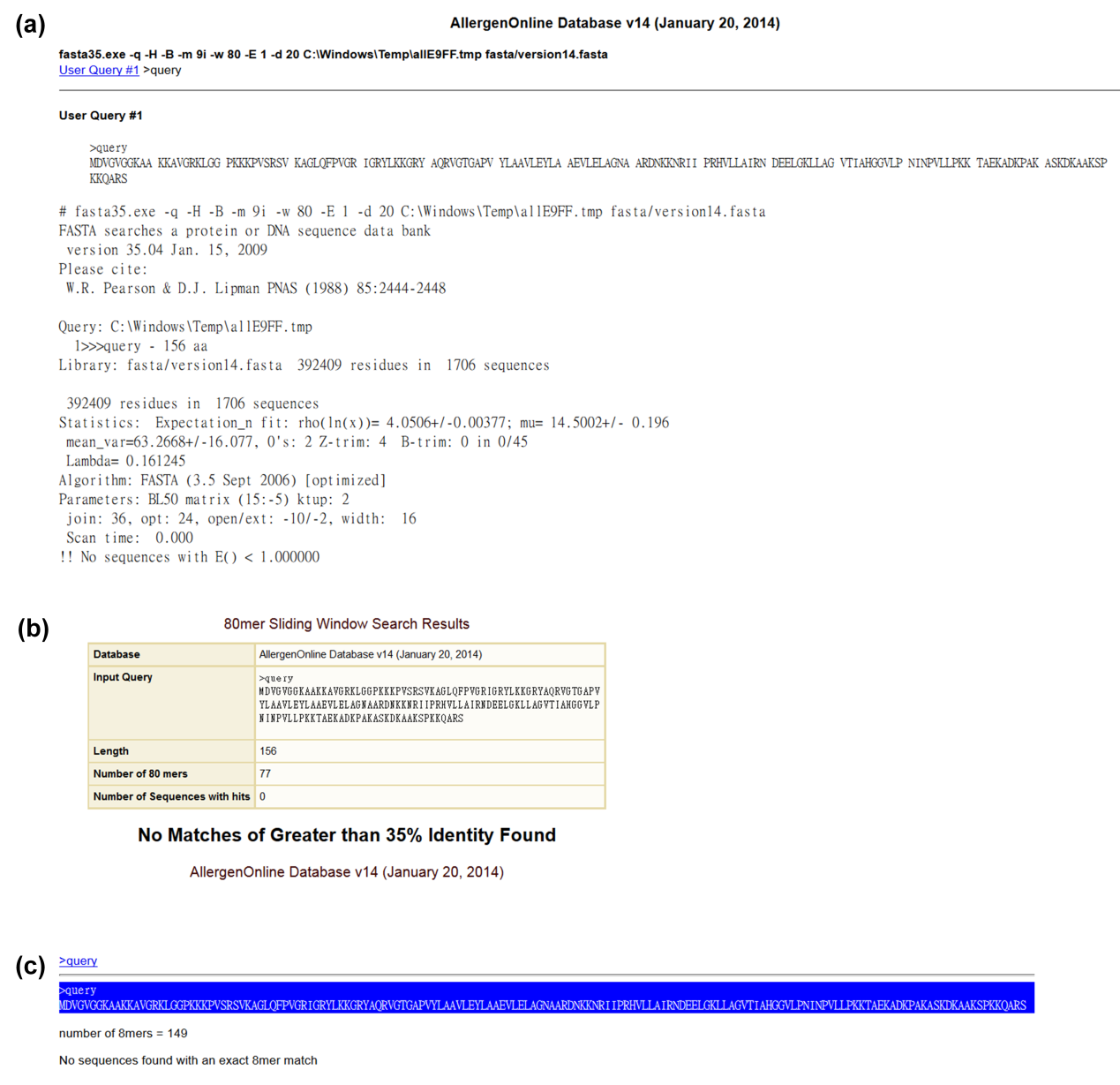


**Supplementary Fig. 2** Homology searches of RLRH1 against allergens in the AllergenOnline database. **a** FASTA search when E-value cutoff = 1. **b** 80-mer search. **c** 8-mer search


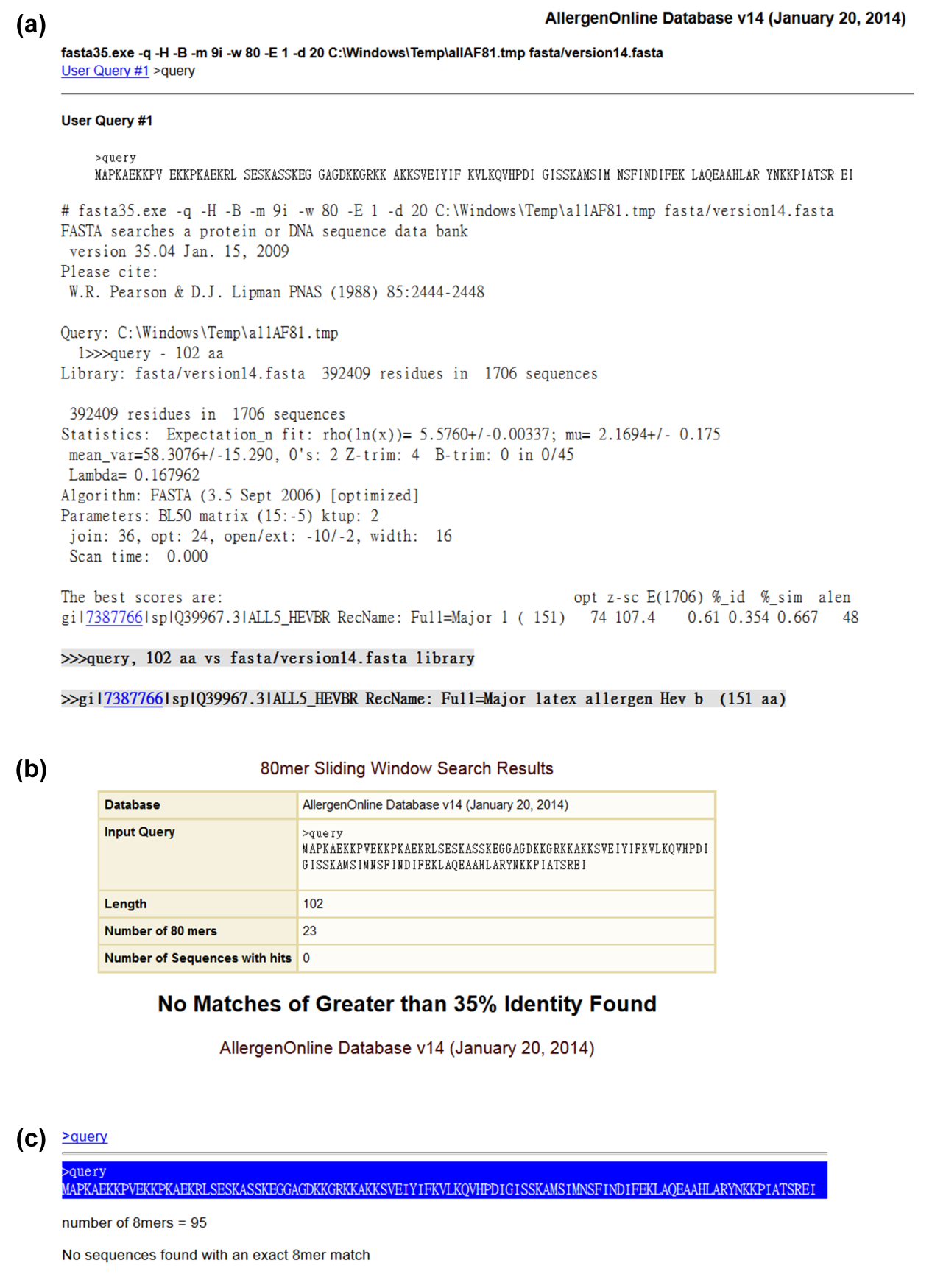


**Supplementary Fig. 3** Homology searches of RLRH2 against allergens in the AllergenOnline database. **a** FASTA search when E-value cutoff = 1. **b** 80-mer search. **c** 8-mer search


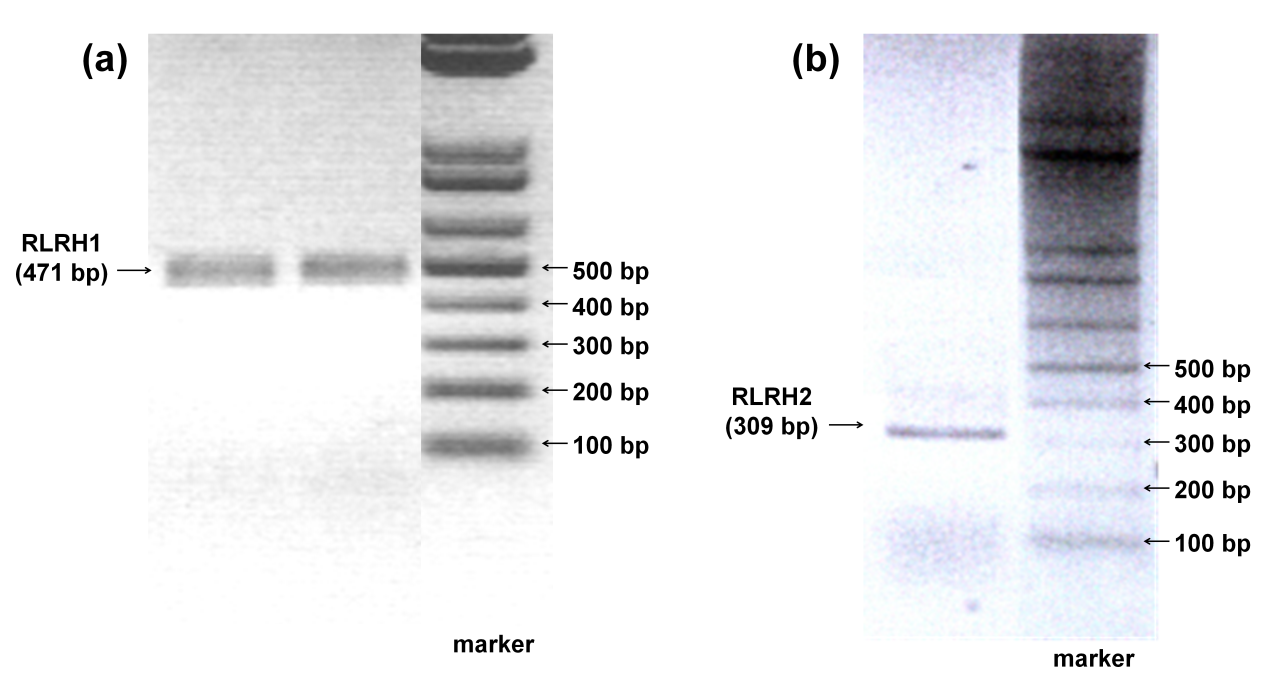


**Supplementary Fig. 4** Cloning of *RLRH1* (**a**) and *RLRH2* (**b**) cDNA by RT-PCR


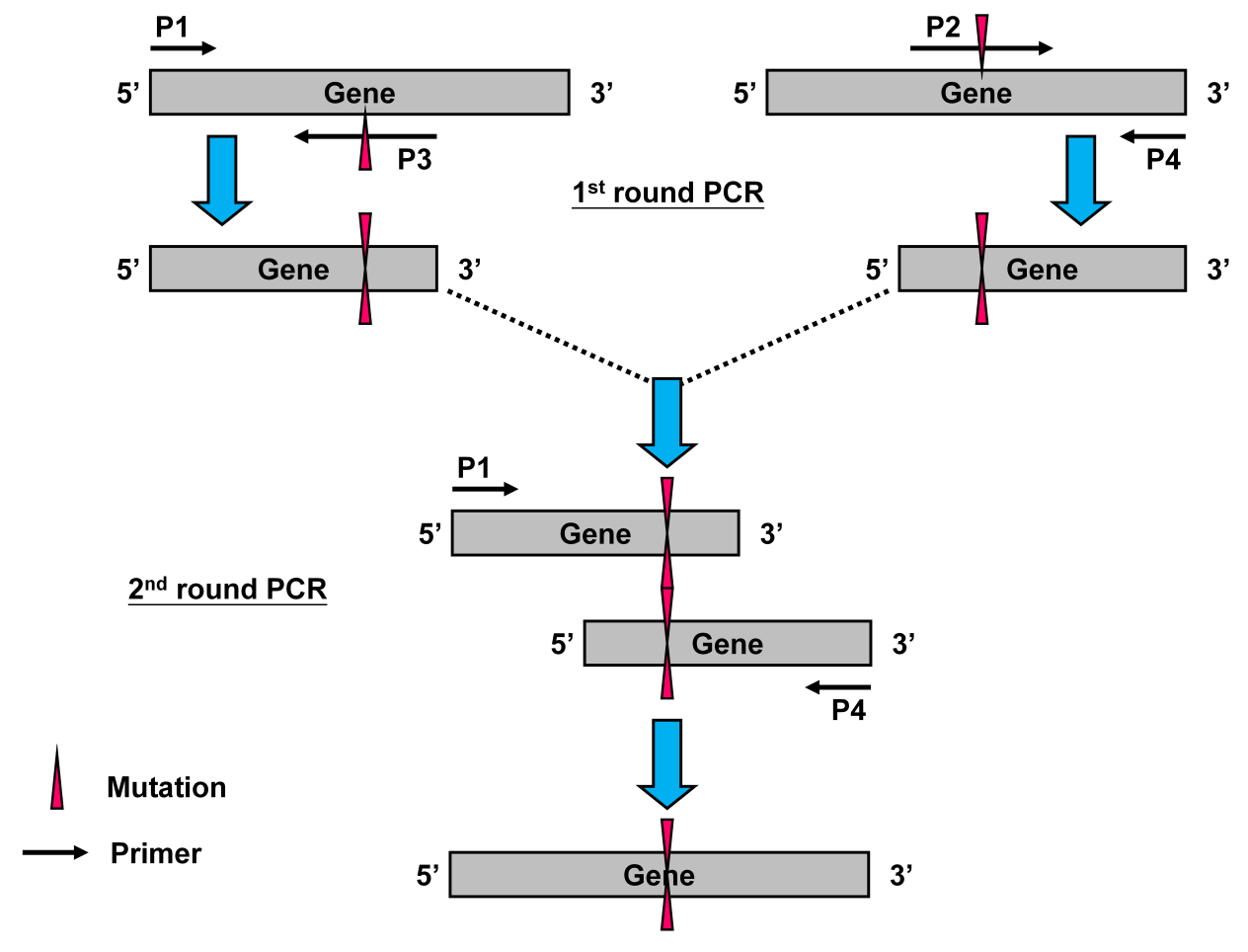


**Supplementary Fig. 5** Procedures used to modify the RLRH1 and RLRH2 amino acid sequences to remove the NLS function. Primers P2 and P3 are complementary and contain modified sequences coded to replace the target amino acids with alanine or glycine in the predicted NLS


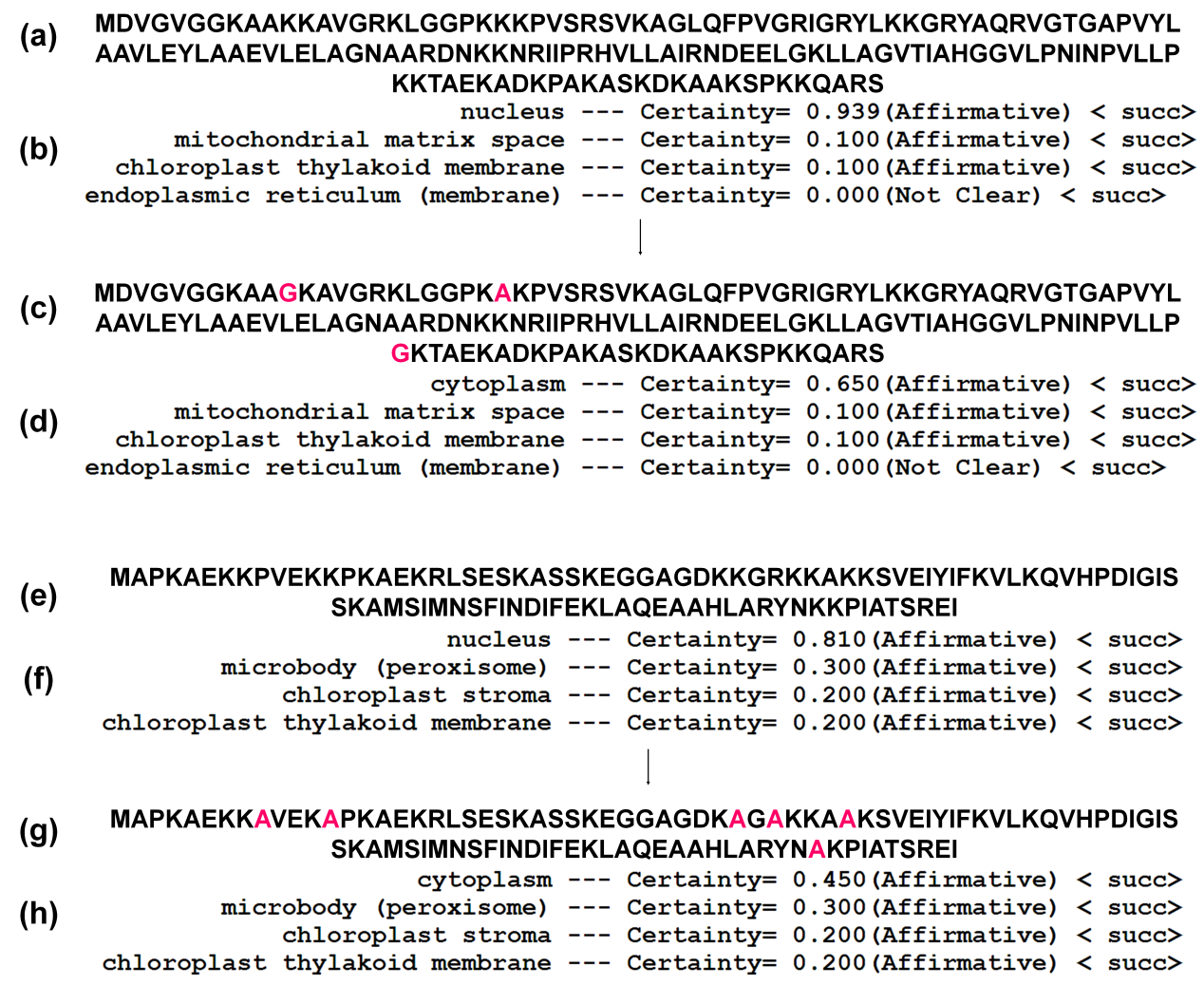


**Supplementary Fig. 6** Predicted nuclear localization signals in RLRH1 and RLRH2 proteins. **a** Amino acid sequence of RLRH1. **b** Predicted subcellular localization of RLRH1 using the PSORT tool. **c** Modified amino acid sequence of RLRH1 (modified amino acids in red). **d** Predicted subcellular localization of modified RLRH1. **e** Amino acid sequence of RLRH2. **f** Predicted subcellular localization of RLRH2. **g** Modified amino acid sequence of RLRH2 (modified amino acids in red). And, **h** predicted subcellular localization of modified RLRH2


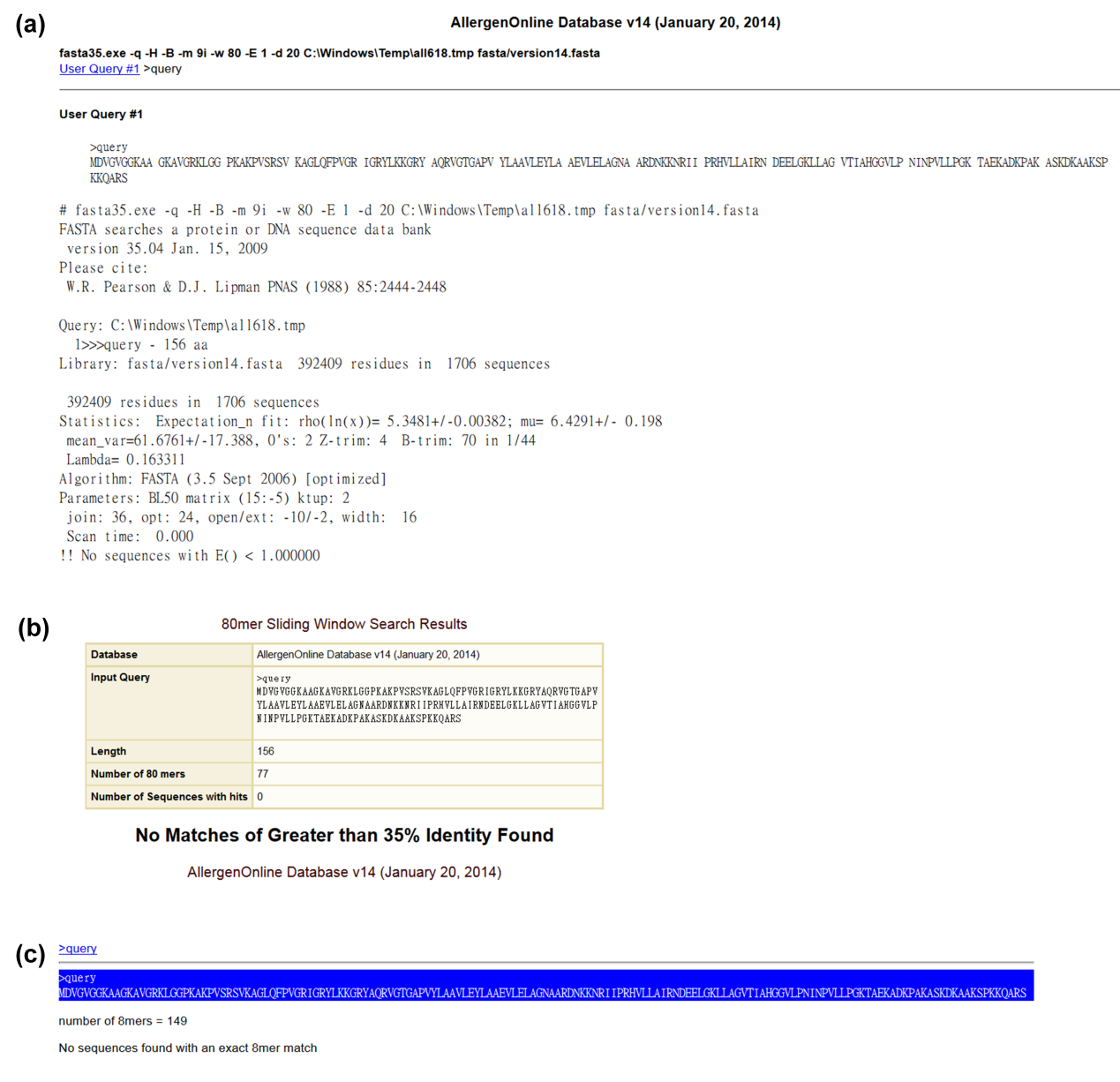


**Supplementary Fig. 7** Homology searches of RLRH1-NLS against allergens in the AllergenOnline database. **a** FASTA search when E-value cutoff = 1. **b** 80-mer search. **c** 8-mer search


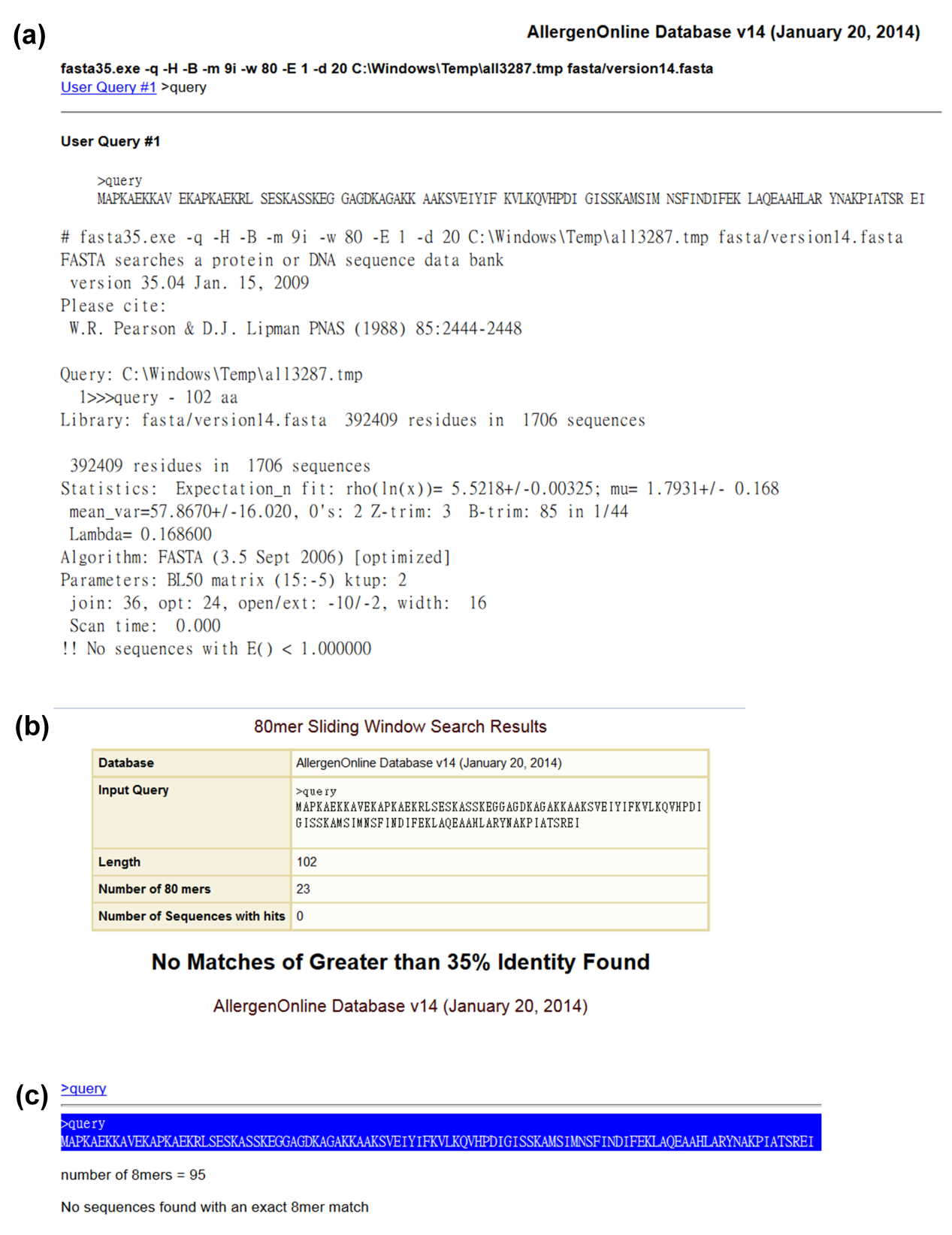


**Supplementary Fig. 8** Homology searches of RLRH2-NLS against allergens in the AllergenOnline database. **a** FASTA search when E-value cutoff = 1. **b** 80-mer search. **c** 8-mer search

**

Supplementary Fig. 9** Southern blot analysis of HLG and LLG transgenic lines. Bands showing successful gene integration (black arrows) were found in LLG and HLG lines in each construct. Sizes were labeled by DIG-labeled DNA marker III (Roche).


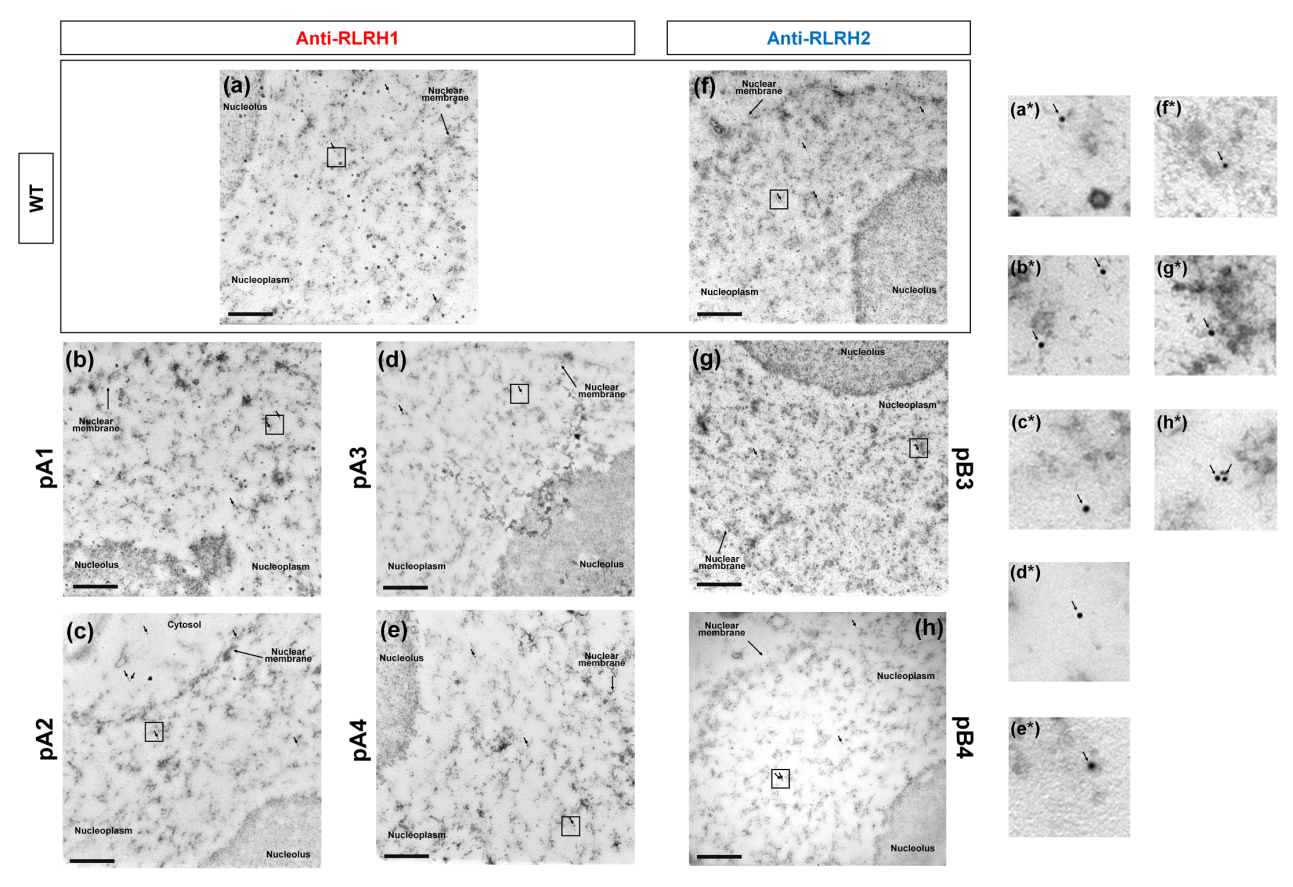
**Supplementary Fig. 10** Background expression of RLRH1 and RLRH2 proteins in aleurone nuclei of WT and transgenic plants visualized by TEM. Immature seeds from WT (**a** & **f**), pA1 (**b**), pA2 (**c**), pA3 (**d**), pA4 (**e**), pB3 (**g**), and pB4 (**h**) were harvested at 10 DAF. Black arrows indicate immunolabeling of the corresponding protein. Bar, 500 nm. Images labeled with asterisks are the enlargements of the corresponding panels


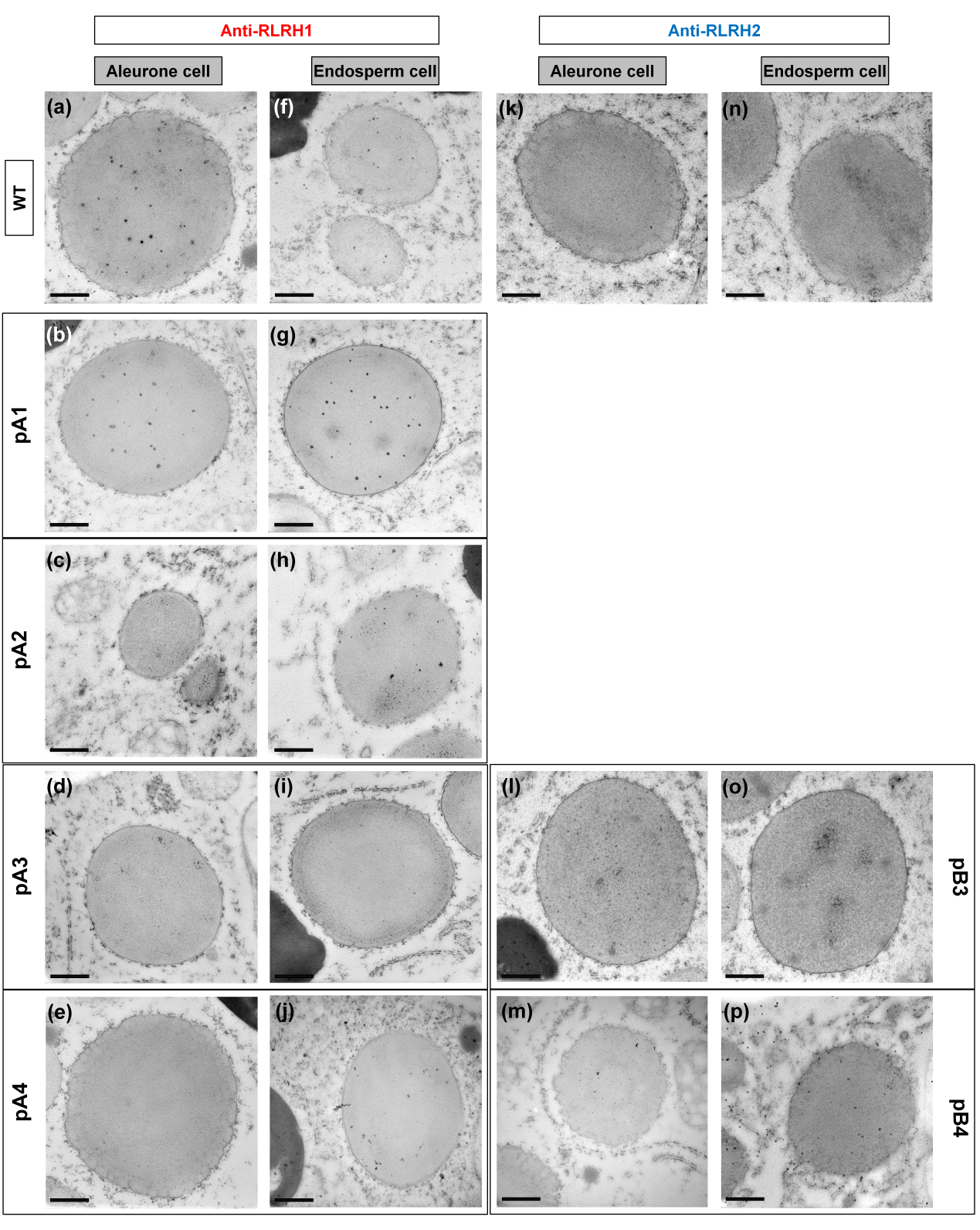


**Supplementary Fig. 11** Morphology of PBs in WT and transgenic plants. **a**, **f**, **k**, and **n**: PBs in WT aleurone and endosperm cells. **b** and **g**: PBs in aleurone and endosperm cells of pA1. **c** and **h**: PBs in aleurone and endosperm cells of pA2. **d** and **i**: PBs in aleurone and endosperm cells of pA3. **e** and **j**: PBs in aleurone and endosperm cells of pA4. **l** and **o**: PBs in aleurone and endosperm cells of pB3. **m** and **p**: PBs in aleurone and endosperm cells of pB4. Bar, 500 nm


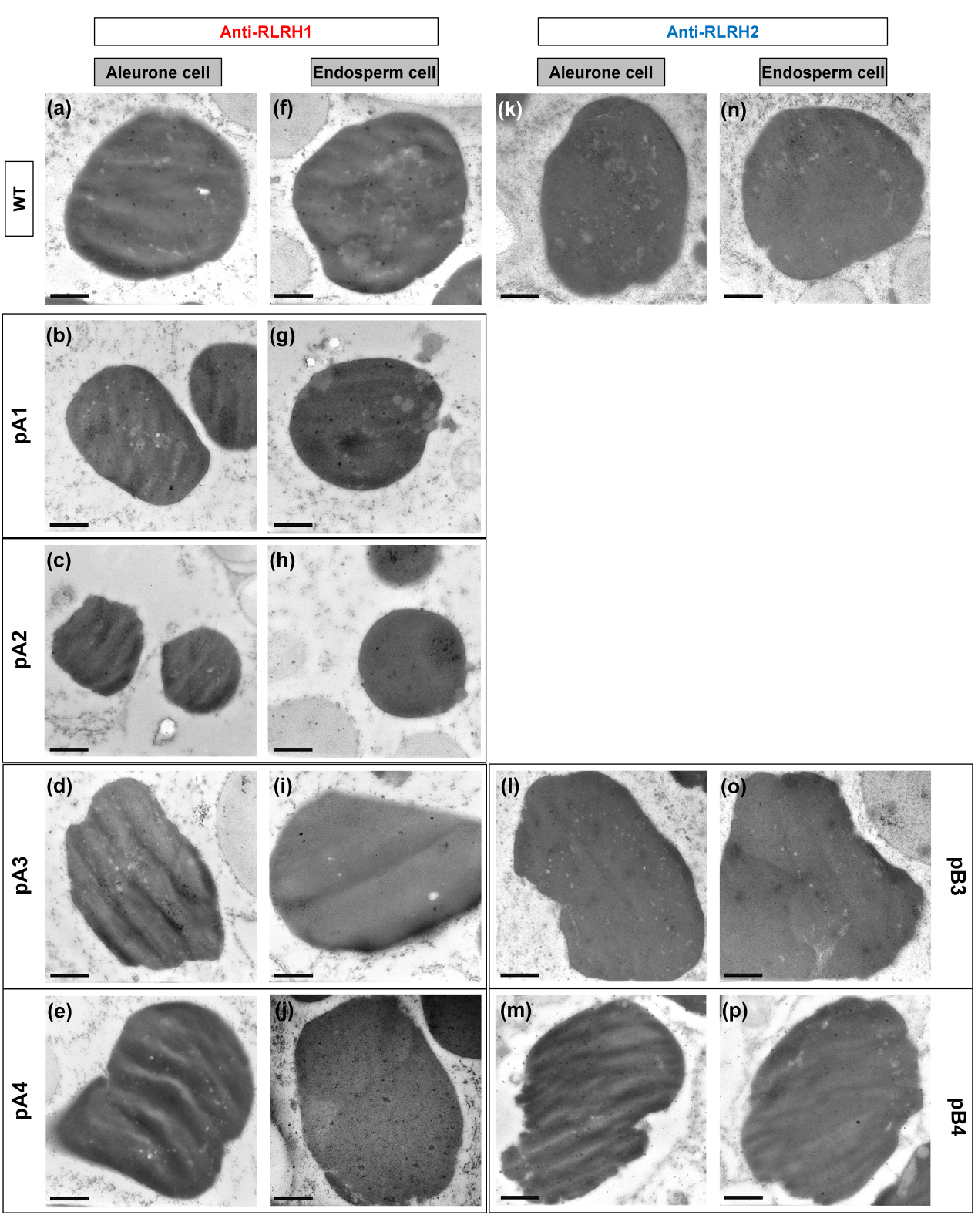


**Supplementary Fig. 12** Morphology of PSVs in WT and transgenic plants. **a**, **f**, **k**, and **n**: PBs in WT aleurone and endosperm cells. **b** and **g**: PBs in aleurone and endosperm cells of pA1. **c** and **h**: PBs in aleurone and endosperm cells of pA2. **d** and **i**: PBs in aleurone and endosperm cells of pA3. **e** and **j**: PBs in aleurone and endosperm cells of pA4. **l** and **o**: PBs in aleurone and endosperm cells of pB3. **m** and **p**: PBs in aleurone and endosperm cells of pB4. Bar, 500 nm


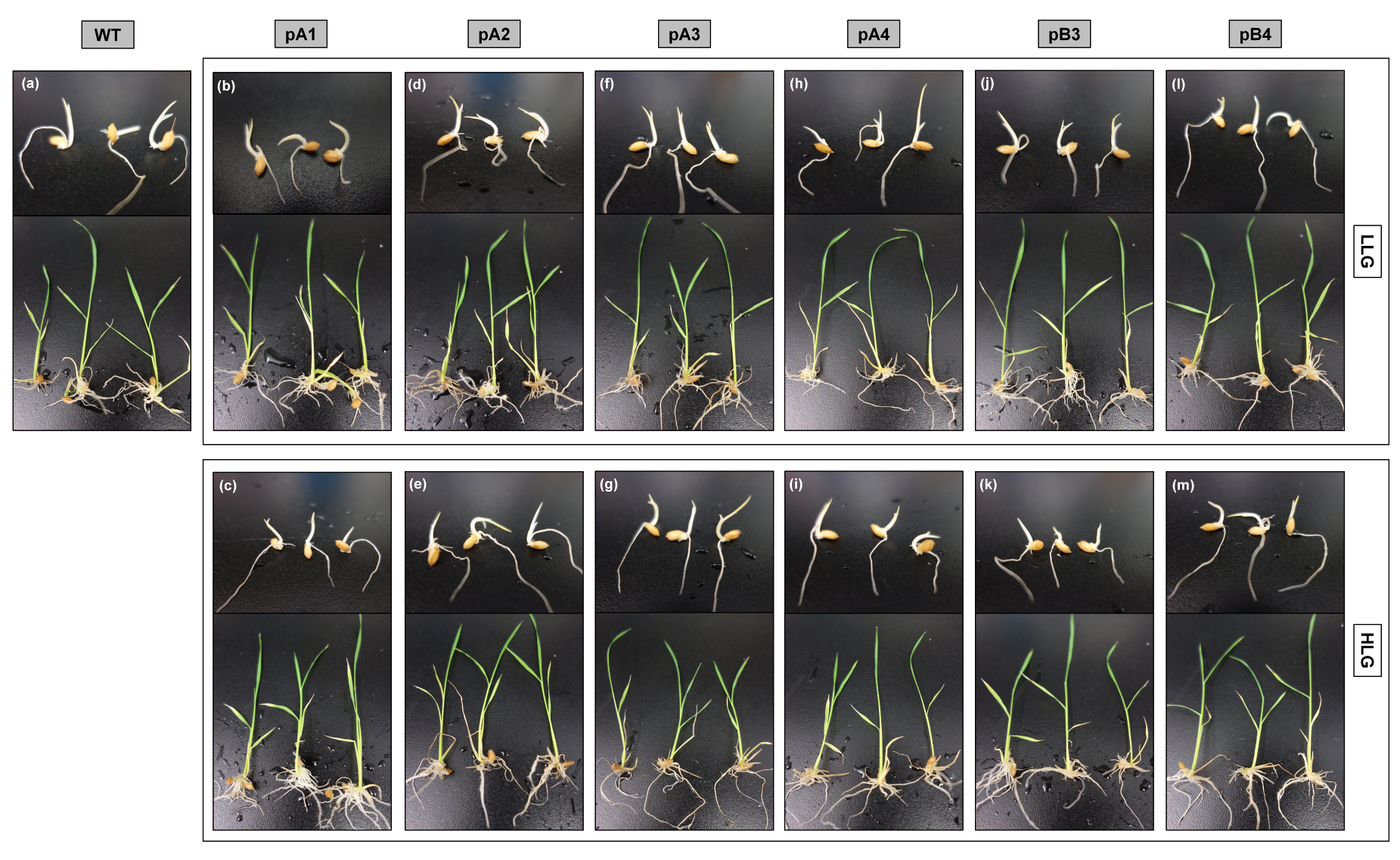


**Supplementary Fig. 13** Morphology of T2 seedlings. WT and T2 seeds from HLG and LLG harboring different constructs were randomly picked and placed in Petri dishes filled with water for germination at 37°C for 5 days. Seedlings were grown for an additional 15 days in Yoshida solution and 8 h/day light conditions. Three seedlings were randomly picked from the WT and each selected transgenic line on the 5^th^ and 20^th^ days after germination to compare morphologies.

**Supplementary Table 1** Summary of the immunolabeling signals of RLRH1 and RLRH2 detected in different subcellular locations in aleurone and endosperm cells of different transgenic rice lines. The “−” sign means that no signal was detected.

| **Cell** | **Aleurone** | | | | | **Endosperm** | | | |
| --- | --- | --- | --- | --- | --- | --- | --- | --- | --- |
| **Organelle** | **Nucleus** | **Cytosol** | **PBs** | **PSVs** | **Others** | **Cytosol** | **PBs** | **PSVs** | **Others** |
| **WT** | **+** | **−** | **−** | **−** | **−** | **−** | **−** | **−** | **−** |
| **pA1** | **+** | **−** | **−** | **−** | **−** | **−** | **−** | **−** | **−** |
| **pA2** | **+** | **+** | **−** | **−** | **−** | **+** | **−** | **−** | **−** |
| **pA3** | **+** | **−** | **−** | **+** | **−** | **−** | **−** | **+** | **−** |
| **pA4** | **+** | **−** | **−** | **+** | **−** | **−** | **−** | **+** | **−** |
| **pB3** | **+** | **−** | **−** | **+** | **−** | **−** | **−** | **+** | **−** |
| **pB4** | **+** | **−** | **−** | **+** | **−** | **−** | **−** | **+** | **−** |

**Supplementary Table 2** Germination rates of T2 transgenic seeds harboring different constructs. T2 seeds were randomly selected and placed in Petri dishes filled with water for germination at 37°C. Germinated seeds were counted on the 5^th^ day, and germination rates were calculated and compared with the WT

|  | **Construct** | **Seeds sown** | **Seeds germinated** | **Germination rate** |
| --- | --- | --- | --- | --- |
|  | **WT** | **40** | **36** | **90.00** |
| **LLG** | **pA1** | **36** | **34** | **94.44** |
|  | **pA2** | **24** | **23** | **95.83** |
|  | **pA3** | **40** | **40** | **100.00** |
|  | **pA4** | **33** | **32** | **96.97** |
|  | **pB3** | **36** | **36** | **100.00** |
|  | **pB4** | **29** | **27** | **93.10** |
| **HLG** | **pA1** | **30** | **30** | **100.00** |
|  | **pA2** | **43** | **43** | **100.00** |
|  | **pA3** | **33** | **32** | **96.97** |
|  | **pA4** | **33** | **33** | **100.00** |
|  | **pB3** | **44** | **42** | **95.45** |
|  | **pB4** | **51** | **49** | **96.08** |
